# ﻿Endemism patterns of the vascular flora of Lebanon: A dynamic checklist

**DOI:** 10.3897/phytokeys.260.156938

**Published:** 2025-07-28

**Authors:** Hicham El Zein, Mauro Fois, Benedetta Gori, Gianluigi Bacchetta

**Affiliations:** 1 Biodiversity Conservation Centre (CCB), Department of Life and Environmental Sciences, University of Cagliari, Cagliari, 09123, Italy University of Cagliari Cagliari Italy

**Keywords:** Biodiversity, Chorotype, IUCN Red List, Levant, Mediterranean, Southwest Asia

## Abstract

Given its high concentration of rare and endangered plant species, Lebanon is recognized as a biodiversity meso-hotspot within the Levant. This study presents a list of vascular plants endemic to Lebanon, detailing their taxonomic diversity, comparing them with the floras of Egypt, Iran and Turkey, and examining their life forms, spatial distribution across key geomorphological features, and conservation status. The list, comprising 169 taxa belonging to 37 families and 99 genera, was compiled through a comprehensive review of published literature, examination of herbarium specimens, and insights from field observations. The five most endemic-rich families are Asteraceae (18.9%), Lamiaceae (14.2%), Fabaceae (11.2%), Caryophyllaceae (5.9%), and Iridaceae (5.9%). The most endemic-rich genus is *Astragalus* (8.3%), followed by *Centaurea* (4.7%), *Allium* (4.1%), and *Iris* (3%). The comparison with other floras highlighted the relationships with the neighboring floristic regions, mostly the Irano-Turanian and Mediterranean. In terms of spatial distribution, Mount Lebanon and Anti-Lebanon emerge as centers of endemism, hosting respectively 70 and 21 exclusive taxa. Hemicryptophytes are the predominant life form (67.6%), followed by geophytes (13.6%), and chamaephytes (10%), reflecting the mountainous and Mediterranean character of Lebanon. According to IUCN Red List, 53% of the taxa have been assessed, with 10% taxa classified as Critically endangered, 27.8% as Endangered, 9.5% as Vulnerable. The checklist is available on an online repository and is considered dynamic. It will be updated in response to taxonomic changes resulting from genetic analyses and revisions of distribution ranges.

## ﻿Introduction

Endemism refers to the exclusive occurrence of an organism within a specific geographic area, which can range from a relatively small region, such as a mountain summit, to a much larger area, such as a continent (Major 1988; Fattorini 2017). The presence of endemic plants in a region is highly regarded from both botanical and biogeographical perspectives, as conservation efforts usually aim at preserving rare and threatened species with limited distributions (Caldecott et al. 1996). Some regions exhibit higher levels of endemism due to the combined influence of environmental conditions and biological processes (Hobohm 2014). The concentrated presence of rare, unique, and threatened species within a geographical area serves at defining global biodiversity hotspots, such as the Mediterranean mega-hotspot (Myers et al. 2000; Cañadas et al. 2014) ranked as the third most important for plant diversity (Lopez-Alvarado and Farris 2022). Located in the Eastern Mediterranean, Lebanon is part of the Levant macro-hotspot (Médail and Quézel 1997, 1999; Médail and Diadema 2009). Previous studies have demonstrated that plant endemism in Lebanon is concentrated within its three mountain ranges, Mount Lebanon, Anti-Lebanon and Mount Hermon, qualifying them as Important Plant Areas (IPAs) and Key Biodiversity Areas (KBAs) for plants (Bou Dagher-Kharrat et al. 2018; El Zein et al. 2018).

**Figure 1.**
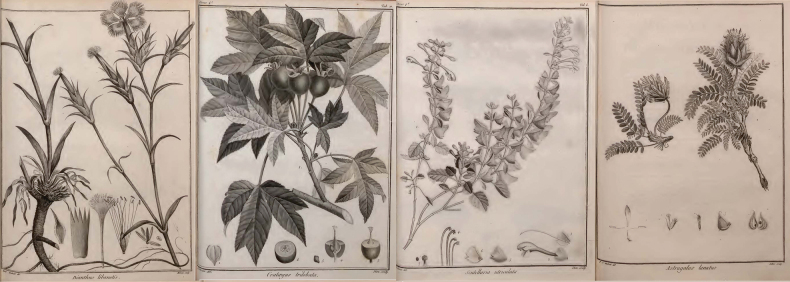
Selection of four drawings from “Icones plantarum Syriæ rariorum” (Labillardière 1791), representing from left to right: *Dianthuslibanotis* Labill., *Malustrilobata* (Labill. ex Poir.) C.K.Schneid., *Scutellariautriculata* Labill., and *Astragaluslanatus* Labill.

The earliest herbarium specimens from the Levant, including present-day Lebanon, Palestine/Israel and Syria, were collected by Rauwolf between 1573 and 1575 (Ghorbani et al. 2018; Stefanaki et al. 2021), and formed the bases for the publication of the first “Flora Orientalis”, which included the mountainous regions of Lebanon (Gronovius and Rauwolf 1755). It was followed by “Flora Palaestina” (Linné and Strand 1759), which included Egypt, Lebanon, and Palestine/Israel. “Icones plantarum Syriæ rariorum” (Labillardière 1791) was the first work exclusively listing plant species within the mountain ranges of Lebanon and Syria with accurate drawings (Fig. 1). At the beginning of the nineteenth century, various botanists made significant contributions to herbaria collections, enhancing the understanding of floral diversity, such as Aucher Eloy, Theodor Kotschy, Isidor Blanche, Charles Gaillardot, Edmont Peyron, Louis Charles Émile Lortet. The second “Flora Orientalis” (Boissier 1867), one of the most remarkable works, provided a list of species occurring from Greece to Afghanistan, including the Levant. Later George Edward Post, published “Flora of Syria, Palestine and Sinaï” (Post 1896), including most regions of the Levant. The twentieth century saw a significant improvement in the quality of botanical publications and an increase in number, particularly with the “Zur flora des Libanon und Antilibanon” (Bornmüller 1914), the “Flore du Liban et de la Syrie” (Bouloumoy 1930), the improved reedition of “Flora of Syria, Palestine and Sinaï” (Post and Dinsmore 1932). Following his explorations with René Gombault and Frère Joseph-Louis, Joseph Thiébaut publishes several notes about the Lebanese and Syrian flora, as well as the “Flore Libano-Syrienne” in three volumes (Thiébaut 1934, 1935, 1937, 1948; Thiébaut and Jarrier 1936, 1940, 1953). After the publication of several volumes of “Zur Flora von Syrien, Libanon und den angrenzenden türkischen Gebieten” (Rechinger 1960), Paul Mouterde published the “Nouvelle Flore du Liban et de la Syrie” which, until now, stands as the most comprehensive reference for the Lebanese flora (Mouterde 1966, 1970, 1984). Finally, Moustapha Nehme (Nehme 1980), and then Georges and Henriette Tohmé (Tohmé and Tohmé 2014), published the first illustrated books with common names in English and Arabic, making botanical knowledge accessible to a wider audience.

Due to substantial taxonomic revisions and recent discoveries in neighboring countries, the list of plant taxa currently considered endemic to Lebanon has undergone changes. Adopting standardized reference frameworks is essential for gaining a deeper understanding of species distribution in the Levant and effectively managing conservation efforts. This study proposes a dynamic checklist on the plant taxa endemic to Lebanon, allowing for continuous updates to reflect changes in distribution driven by genetic analyses and newly documented occurrences. To establish a more coherent biogeographic framework on the Levantine flora, a geomorphological approach was adopted instead of relying on strict administrative boundaries. The flora of Anti-Lebanon and Mount Hermon, whose ranges extend across borders with neighboring countries, was entirely considered. The taxonomic patterns of endemism identified in Lebanon are then compared with those of Egypt, Iran and Turkey, to gain further insights into their relationships with the Mediterranean, Irano-Turanian, and Saharo-Arabian biogeographic regions represented by these countries (Zohary 1973; Takhtajan 1986). The proportions of life forms among endemic taxa are calculated, and their potential adaptive strategies are discussed. Finally, extinction risks for the selected taxa are presented to assess the level of threat Lebanon’s endemic flora is currently facing.

## ﻿Material and methods

### ﻿Study area

Lebanon is one of the most mountainous countries in the world, with 81% of its area (10,452 km^2^) covered by mountains with an average elevation of 1,250 meters above sea level (asl) (Fayad et al. 2017; World Population Review 2025). Three mountain ranges, namely Mount Lebanon, Anti-Lebanon and Mount Hermon, run parallel to the coast of the Mediterranean Sea, and are oriented South-southwest and North-northeast, on a distance of approximately 170 km (Dubertret 1941, 1945; Vaumas 1954; Sanlaville 1969). Mount Lebanon is the westernmost range and is located in direct contact with the sea. It rises in south Lebanon and extends northwards to the Homs Gap, a flattened area that separates it from the coastal Syrian range (Suriano 2013). Anti-Lebanon and Mount Hermon, located in the east, are geographically aligned and were historically considered part of the same range. Studies have shown that their orogenic processes were different (Walley 1998). The Beqaa is a plateau situated between Mount Lebanon one side, and Anti-Lebanon and Mount Hermon on the other side. With an average elevation of 1,100 m asl, it serves as a natural corridor between the ranges and is the only extensive flat area in the country (Sanlaville 1963). Mount Lebanon, reaching an elevation of approximately 3,088 m asl, acts as an orographic barrier, intercepting most of the clouds (Heybroek 1942). As a result, its western slopes, facing the sea, receive higher precipitations, whereas its eastern slopes, along with the Beqaa and Anti-Lebanon, experience drier conditions due to the rain shadow effect (Vaumas 1954; Baldy 1959; Jomaa et al. 2019). The borders of Lebanon entirely encompass Mount Lebanon, which extends northward from Akkar Governorate to Jezzine District southward. Anti-Lebanon is located between Lebanon and Syria, with the borders between these two countries generally following the crest of the range. A similar situation applies to Mount Hermon, except that the southern slopes of the range, which are part of the Syrian territory, have been occupied by Israel since 1967 (Dar 1988).

## ﻿Taxonomic sources

The “Nouvelle Flore du Liban et de la Syrie” (Mouterde 1966, 1970, 1984) was chosen as reference flora for the study as it is considered the most comprehensive and coherent reference, synthesizing all previous works published during the nineteenth and twentieth centuries and citing most specimens used for species description and taxonomic verification. This reference served as the foundation for compiling the initial endemic list. To ensure the inclusion of all taxa considered endemic, five previous floras (Labillardière 1791; Boissier 1867; Post 1896; Bouloumoy 1930; Post and Dinsmore 1932) and the “Illustrated Flora of Lebanon” (Tohmé and Tohmé 2014) were also consulted. The total number of plant taxa in Lebanon is estimated at approximately 2,652, including the 2,612 taxa proposed in (Tohmé and Tohmé 2014), along with 40 newly reported occurrences from the latest publications (Williams et al. 2015; Addam et al. 2019, 2020; El Zein and Bou Dagher-Kharrat 2021; El Zein and Pouzoulet 2021; El Zein et al. 2023; Maalouf et al. 2024; Maalouf and Binzet 2025).

Following an exhaustive and detailed review of the reference flora, the initial list was compiled. Each taxon was systematically verified to assess any change in distribution or taxonomy treatment, by examining recent works on the flora of the neighbouring regions, such as Cyprus (Meikle 1977), Egypt (Muschler 1912; Boulos 1999; Bedair et al. 2023), Iraq (Townsend and Guest 1974; Ghazanfar et al. 2013, 2019; Ghazanfar and Edmondson 2016), Jordan (Taifour and El-Oqlah 2017), Palestine/Israel (Zohary 1966, 1972; Feinbrun-Dothan 1978, 1986; Danin and Fragman-Sapir 2016), and Turkey (Davis et al. 1965, 1967, 1970, 1972, 1975, 1978, 1982, 1984, 1985, 1988; Güner et al. 2000). When available, studies on specific taxonomic groups were reviewed to detect any changes, such as those on Pteridophytes (Fraser-Jenkins and Parris 2021), *Allium* (Brullo et al. 1996; Fritsch et al. 2010; Fragman-Sapir and Fritsch 2011), *Alyssum* (Španiel et al. 2015), *Chaerophyllum* (Piwczyński et al. 2015), *Cousinia* (Mehregan and Kadereit 2008), *Dichoropetalum* (Pimenov et al. 2007), *Erysimum* (Polatschek 2010), *Ferulago* (Bernardi 1979), *Johrenia* (Pimenov et al. 2007), *Noccaea* (Al-Shehbaz 2014), *Papaver* (Kadereit 1993), *Pimpinella* (Fereidounfar et al. 2016), *Thlaspi* (German 2018), *Tulipa* (Christenhusz et al. 2013), and *Vicia* (Jalilian et al. 2014). Herbarium specimens, including types, where available, were provided by the Herbaria of Geneva (G; CHG, 2025), Kew (K; RBGK, 2025), Edinburgh (E; RBGE, 2025), Paris (P; MNHN, 2025) and Wien (W; NHM, 2025). Global online databases on plant taxonomy were used to verify any changes in nomenclature and distribution for each taxon (Euro+Med 2025; POWO 2025).

### ﻿Selection criteria for endemism

**Figure 2.**
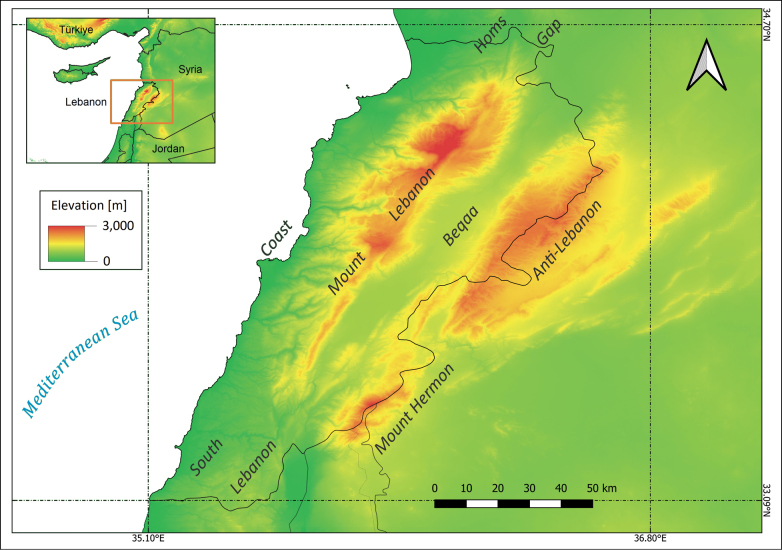
Topographic map of the seven main geographic regions of Lebanon.

The checklist included taxa that are exclusive endemic to one, or more, of the seven major geomorphological features found, fully or partially, included within Lebanon, namely Anti-Lebanon range, Beqaa, the coast with the Mediterranean Sea, Homs Gap, Mount Hermon, Mount Lebanon, and South Lebanon (Fig. 2). Endemism was only considered at the species and subspecies level, with endemic varieties and forms excluded from this checklist.

Finally, following our review, each taxon of the initial list was assigned to one of three categories, namely included (includenda), excluded (excludenda), or doubtful (inquirenda), as proposed in the checklist of endemic plants of Sardinia (Fois et al. 2022). Taxa with valid taxonomic status and confirmed distribution, exclusive to the above-mentioned seven regions, were considered endemic and included in the final checklist. Taxa with broader distributions, extending outside the defined area, those synonymized with taxa of wider distribution, or inadequately described, were excluded from the checklist as they no longer met the criteria for endemism. Taxa with uncertain taxonomy were classified as doubtful and not retained in the final checklist, as they require further investigations, particularly genetic analyses, to clarify their status.

The taxonomic distribution of the endemic flora of Lebanon was analyzed in terms of families and genera and then compared with that of Egypt, Iran, and Turkey. The taxonomy of the endemic taxa from these three countries was collected from the most recent publications on the floras of Egypt (Abdelaal et al. 2018; Abdelaal 2020), Iran (Noroozi et al. 2019a, 2024; Pahlevani et al. 2020; Safikhani and Mahmoodi 2020; Khalvati and Ranjbar 2021; Asgari et al. 2022; Dolatyari et al. 2023), and Turkey (Aytaç and Duman 2004; Al-Shehbaz et al. 2007; Oybak Dönmez and Işik 2008; Tabur et al. 2009; Akçiçek et al. 2012; Denız et al. 2015; Duran et al. 2015; Karaveliogullari et al. 2016; Uysal et al. 2016; Buira et al. 2017; Kuşaksiz 2019; Noroozi et al. 2019b; Bozyel and Merdamert-Bozyel 2020; Sezer 2021; Koçyiğit et al. 2023).

### ﻿Life forms and conservation statuses

The classification of life forms follows Raunkiær’s system (Raunkiær 1934), based primarily on the reference flora and supplemented with insights from field observations.

The conservation statuses, according to the guidelines and criteria of the International Union for Conservation of Nature (IUCN 2024), were added when global assessments were available. The categories were abbreviated as follows: Critically Endangered **CR** = Critically Endangered, **EN** = Endangered, **VU** = Vulnerable, **NT** = Near Threatened, **LC** = Least Concern, **na** = not assessed.

### ﻿Map and figures

The map was developed with QGIS software (QGIS Development Team 2023), using shapefiles available on DIVA-GIS website (DIVA-GIS 2023). Analysis of life forms, extinction risks and phylogenetic relationships between taxa were performed using R software (R Core Team 2024) and associated packages: *ape* (Paradis et al. 2024), *devtools* (Wickham et al. 2022), *dplyr* (Wickham et al. 2023b), *lattice* (Sarkar et al. 2023), *rlang* (Henry and Wickham 2023), and *tidyr* (Wickham et al. 2023a). Figures were built using Rawgraphs (Mauri et al. 2017) and iTOL (Letunic and Bork 2024).

## ﻿Results

### ﻿Included species

**Figure 3.**
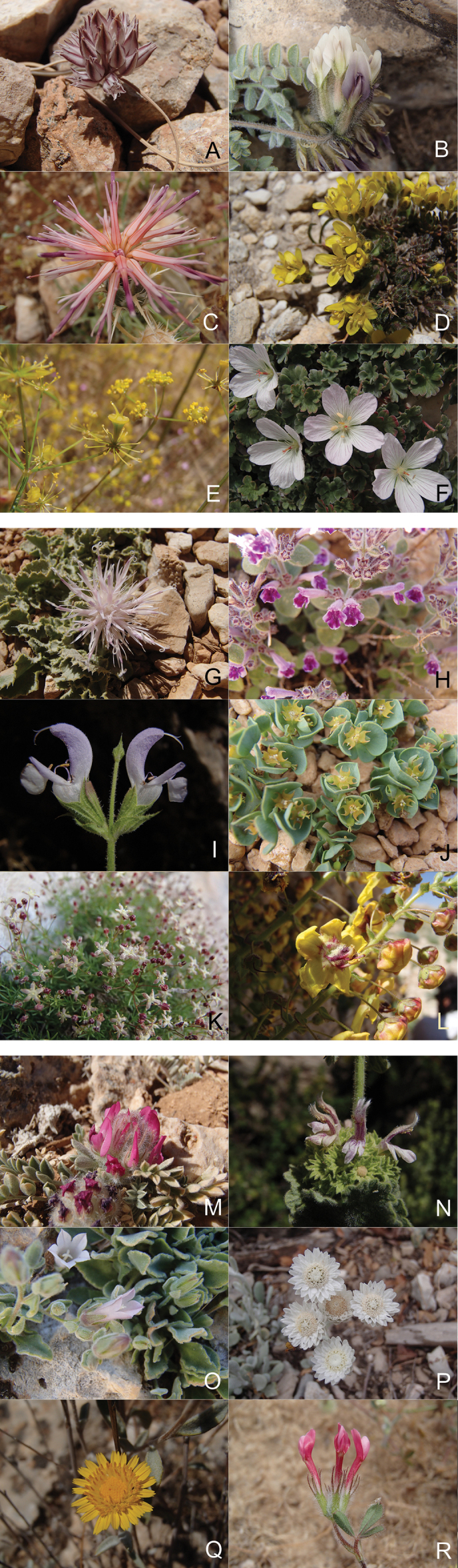
A selection of eighteen iconic plant taxa endemic to Lebanon; **A.***Alliummachmelianum* Post; **B.***Astragaluslanatus* Labill.; **C.***Centaureaainetensis* Boiss.; **D.***Drabaantilibanotica* Al-Shehbaz; **E.***Chaerophyllumaurantiacum* Post; **F.***Geraniummakmelicum* Aedo.; **G.***Myopordonpulchellum* (Winkler & Barbey) Wagenitz; **H.***Clinopodiumlibanoticum* (Boiss.) Kuntze; **I.***Salviajosetta* El Zein.; **J.***Euphorbiacaudiculosa* Boiss.; **K.***Galiumjungermannioides* Boiss.; **L.***Verbascumantilibanoticum* Hub.-Mor.; **M.***Astragaluskurnet-es-saudae* Eig.; **N.***Ballotaantilibanotica* Post; **O.***Campanuladamascena* Labill.; **P.***Helichrysumvirgineum* DC.; **Q.***Rhanteriopsislanuginosa* (DC.) Rauschert; **R.***Trifoliummeduseum* C.I.Blanche ex Boiss.

The checklist includes 169 taxa (Suppl. material 1), representing approximately 6.4% of the entire native flora of Lebanon. This total includes 17 endemic taxa that were reported after the publication of the reference flora. Among these, 15 taxa are new to science and have been described over the past fifty years. *Scillalibanotica* Speta (Speta 1976) and *Alliumhermoneum* Kollmann & Schmida (Kollmann and Schmida 1977) were the earliest to be described, shortly after the publication of the first volume of the reference flora documenting monocotyledons (Mouterde 1966). The other 13 new endemic taxa were described between 2002 and 2025: *Alliumpseudostamineum* Kollmann & Shmida (Brullo et al. 2007), *Ballotabyblensis* Semaan & R.M.Haber (Semaan and Haber 2004), *Crocusbaalbekensis* Addam & Bou-Hamdan (Addam et al. 2019), *Dactylorhizaphoenissa* (B.Baumann & H.Baumann) P.Delforge (Delforge 2015), *Isoeteslibanotica* Musselman, Bolin & R.D.Bray (Bolin et al. 2011), *Limoniummouterdei* Domina, Erben & Raimondo, *L.postii* Domina, Erben & Raimondo (Domina et al. 2008), *Onosmasanninensis* Maalouf & Binzet (Maalouf and Binzet 2025), *Phlomistathamiorum* R.M.Haber & Semaan (Haber and Semaan 2002), *Romuleajezzinis* K.Addam & M.Bou-Hamdan, *R.libanotica* K.Addam & M.Bou-Hamdan (Addam and Bou-Hamdan 2022), *Salviafairuziana* R.M.Haber & Semaan (Haber and Semaan 2004) and *S.josetta* El Zein (El Zein and Bottcher 2024). A taxonomic revision confirmed that the oak species *Quercuskotschyana* O. Schwarz is endemic to Lebanon (Stephan and Teeny 2017). One species, *Irisbasaltica* Dinsm., considered endemic to Homs Gap, was recently discovered in Akkar (Raab-Straube et al. 2020). A photographic selection of 18 iconic endemic taxa is presented (Fig. 3).

### ﻿Doubtful taxa

Nine doubtful taxa, not included in the checklist, are presented here with explanations and references for their uncertain status (Suppl. material 2).

### ﻿Excluded taxa

Of the 207 taxa previously considered as endemic to Lebanon in the reference flora (Mouterde 1966, 1970, 1984), 48 are no longer considered as endemic (Suppl. material 3), as their distribution ranges have extended beyond Lebanon to neighboring countries, including Turkey or Iran. Consequently, these taxa were excluded from the final checklist. Most of them (28) kept their original names and have been recorded in neighboring countries, especially Turkey, or as far as Central Asia. In contrast, 19 taxa underwent taxonomic revisions and were synonymized with taxa present in other countries. Finally, *Althaeabertramii* Post & Beauverd was the only taxon excluded because of misdescription. The sole collected specimen was later identified as *Kitaibeliabalansae* Boiss. (CHG 2025).

### ﻿Taxonomic diversity

**Figure 4.**
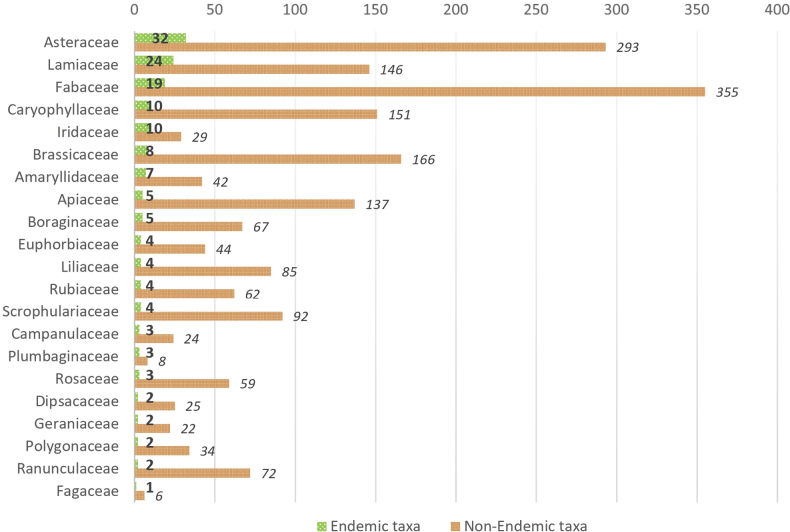
Number of endemic and non-endemic taxa in the 21 most endemic-rich families of the vascular flora of Lebanon.

Plant endemism in Lebanon is only observed at the species level, as there are no endemic families or genera. All endemic taxa are spermatophytes, seed-producing plants, apart from a single species of *Isoetes*, belonging to the lycophytes clade. The 169 endemic taxa are distributed across 37 families. The eight most endemic-rich families are Asteraceae (32), Lamiaceae (24), Fabaceae (19), Caryophyllaceae (10), Iridaceae (10), Brassicaceae (8), Amaryllidaceae (7), and Apiaceae (5) which represent 68% of the endemic flora (Fig. 4). In terms of proportion, five families have a high number of endemic taxa compared to their total species diversity, namely Iridaceae (34.5%), Plumbaginaceae (27.3%), Fagaceae (16.7%), Amaryllidaceae (14.3%) and Lamiaceae (14.1%).

**Figure 5.**
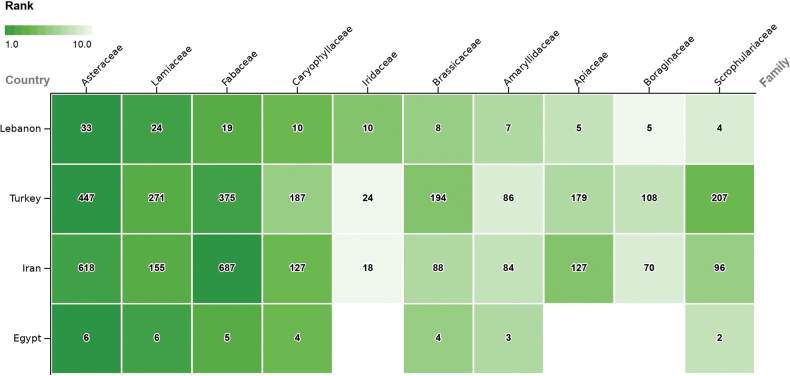
Comparison between the 10 most endemic-rich plant families in Lebanon, with number of taxa, with families in Egypt, Iran and Turkey. Blank cells equal 0, while white cells were not ranked among the top 10 (Sources in Methods).

The ten most endemic-rich families in Lebanon are compared with those of three major neighboring countries, namely Egypt, Iran and Turkey (Fig. 5). Asteraceae, Lamiaceae and Fabaceae constitute the top three most endemic-rich families in the four countries. Iran and Turkey share the same nine most endemic-rich families as Lebanon, but in different order. Only their tenth family, Rosaceae, differs. The major difference is the lower proportion of endemic taxa in Iridaceae, as this family is not included among the 20 most endemic-rich families of Iran and Turkey. Egypt shows a different profile, sharing seven most endemic-rich families with Lebanon, while the remaining three, Asparagaceae (4), Polygonaceae (2), and Solanaceae (1), are not represented among the richest in Lebanon.

**Figure 6.**
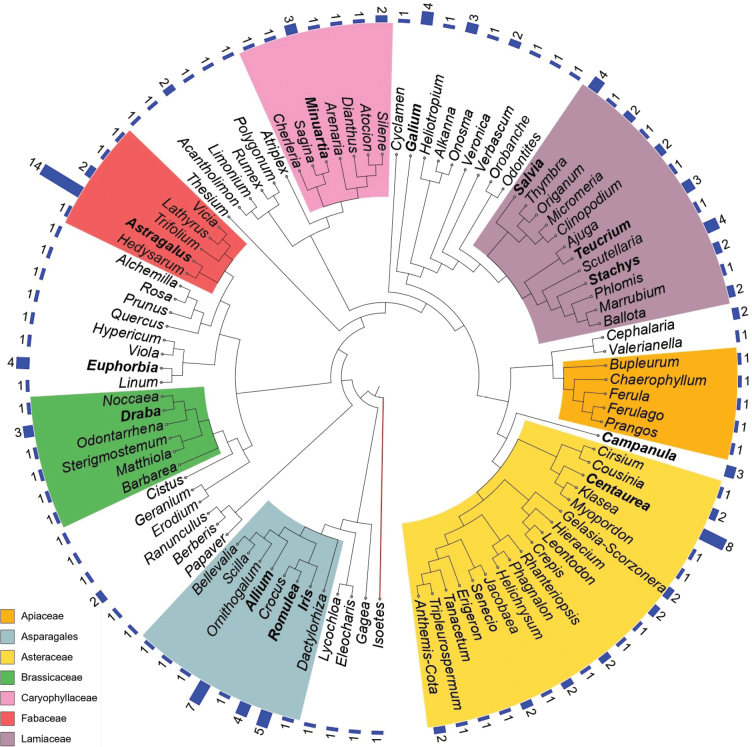
Phylogenetic tree showing the distribution of the plant taxa endemic to Lebanon across genera. Six of the most endemic-rich families are highlighted in color, as well as the order of the Asparagales which gathers the families Amaryllidaceae (*Allium*), Asparagaceae (*Bellevalia*, *Ornithogalum*, *Scilla*), Iridaceae (*Crocus*, *Iris*, *Romulea*), and Orchidaceae (*Dactylorhiza*).

The 169 endemic taxa are distributed across 99 genera (Fig. 6). The fifteen most endemic-rich genera are *Astragalus* (8.3%), *Centaurea* (4.7%), *Allium* (4.1%), *Iris* (3%), *Euphorbia* (2.3%), *Galium* (2.4%), *Romulea* (2.4%), *Salvia* (2.4%), *Stachys* (2.4%), *Alkanna* (1.8%), *Campanula* (1.8%), *Draba* (1.8%), *Minuartia* (1.8%), and *Teucrium* (1.8%), containing 41% of the total number of endemic species. Asteraceae, Lamiaceae, and Caryophyllaceae are the most genera-rich families with respectively 18, 12, and 7 genera.

**Figure 7.**
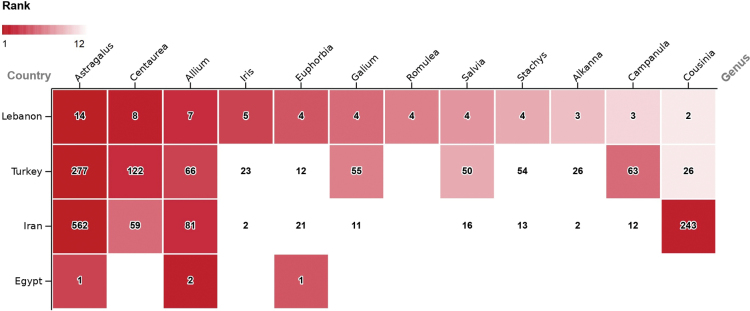
Comparison between the 12 most endemic-rich plant genera in Lebanon, with the number of taxa, with genera in Egypt, Iran and Turkey. Blank cells equal 0, while white cells were not ranked among the top 12 (Sources in Methods).

The twelve genera containing the highest number of endemics in Lebanon are compared with those of Egypt, Iran and Turkey (Fig. 7). In all four countries, *Astragalus* and *Allium* rank among the top four genera. The flora of Turkey shows the most similarities to that of Lebanon, as seven of the twelve most endemic-rich genera are common to both, including *Astragalus*, *Centaurea*, and *Allium*, which all rank among the top four. They are followed by *Galium*, *Salvia*, *Campanula*, and *Cousinia* (Vanderplank et al. 2014). Iran shares four of the twelve most endemic-rich genera, including *Astragalus*, *Centaurea*, *Allium* and *Cousinia*. Egypt shares three families of the Lebanese top 12, including *Astragalus*, *Allium*, and *Euphorbia*.

### ﻿Life forms in endemics

**Figure 8.**
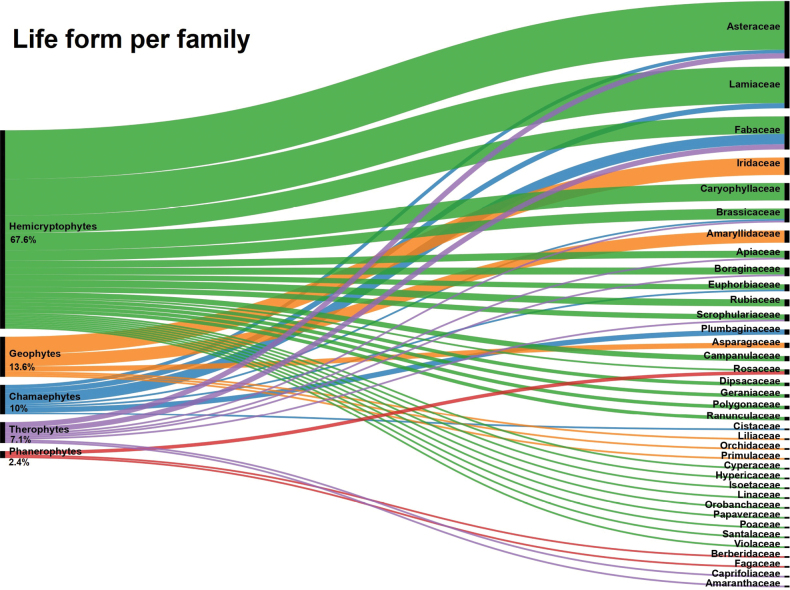
Distribution of Raunkiær’s life forms across families of plant taxa endemic to Lebanon.

The distribution of Raunkiær’s life forms among plant families endemic to Lebanon (Fig. 8) reveals the predominance of hemicryptophytes (67.6%), followed by geophytes (13.6%), chamaephytes (10%), therophytes (7.1%) and phanerophytes (2.4%).

### ﻿Distribution of endemism across Lebanon

**Figure 9.**
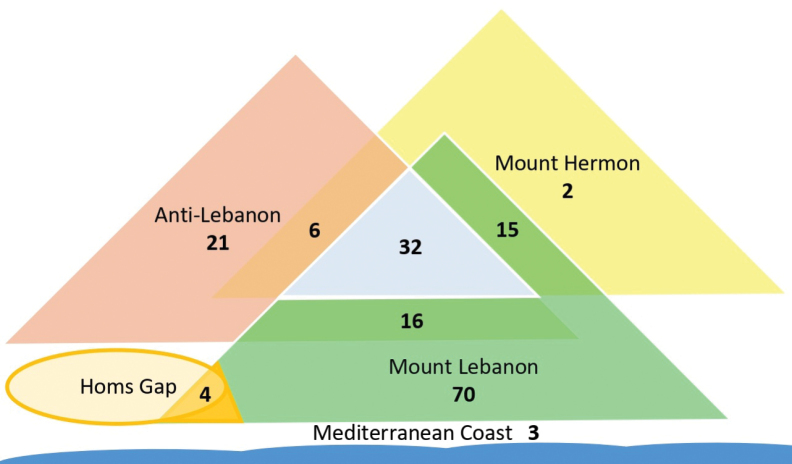
Schematic distribution of endemic plant taxa within and between the regions of Lebanon.

The endemic taxa are distributed across Mount Lebanon, Anti-Lebanon, Mount Hermon, the Mediterranean coast and Homs Gap. Mount Lebanon is the most endemic-rich range, hosting 70 exclusive taxa (41.4%), followed by Anti-Lebanon with 21 taxa (12.4%), the Mediterranean coast with three taxa (1.8%), and Mount Hermon with two taxa (1.2%). Additionally, 16 taxa are shared between Mount Lebanon and Anti-Lebanon, 15 between Mount Lebanon and Mount Hermon, six between Anti-Lebanon and Mount Hermon, four between Mount Lebanon and Homs Gap, and 32 across all three ranges. A simplified representation of the distribution of the endemic taxa within and between the regions is presented (Fig. 9).

### ﻿Conservation statuses

**Figure 10.**
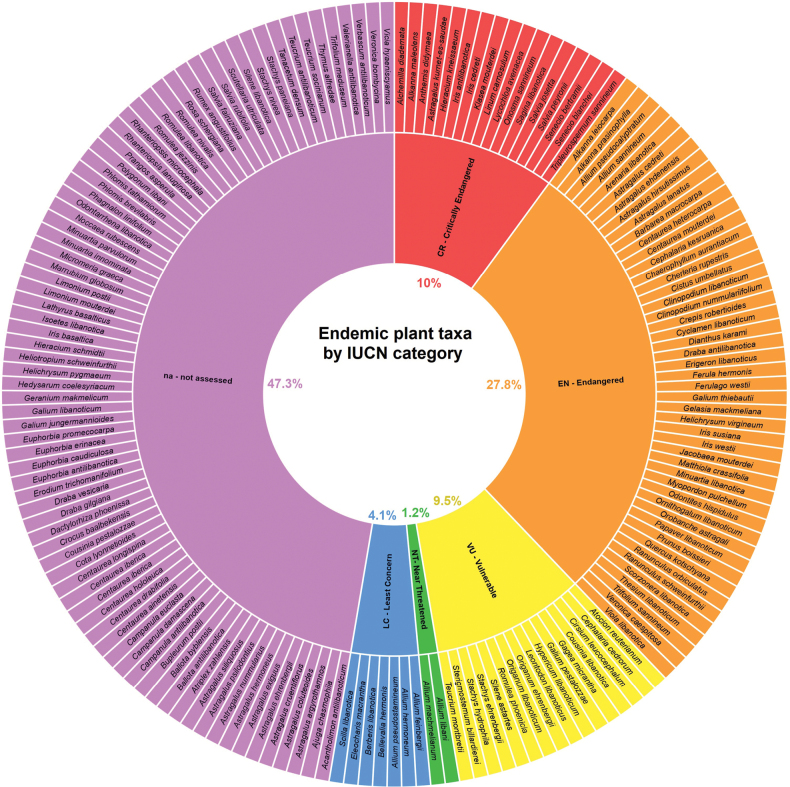
Proportion of the 169 plant taxa endemic to Lebanon that have been assessed and not assessed, along with their respective IUCN Red List categories, CR = Critically Endangered, EN = Endangered, VU = Vulnerable, NT = Near Threatened, LC = Least Concern, na = not assessed.

Approximately 53% of the endemic taxa were assessed according to IUCN guidelines and criteria (Fig. 10). Among them, 17 were evaluated as Critically endangered (10%), 47 as EN (27.8%), 16 as VU (9.5%), two as NT (1.2%), seven as LC (4.1%). Eighty were not assessed.

## ﻿Discussion

Among the 169 taxa, six with well-documented descriptions and multiple collected specimens, but not observed for over 50 years, are included in the final list (Suppl. material 1) as their historical presence was exhaustively documented, leading to the high probability that they are still extant: *Alkannamaleolens* Bornm., *Erophilagilgiana* (Muschler) O.E.Schultz, *Klaseamouterdei* (Arènes) Greuter & Wagenitz, *Odontarrhenalibanotica* (Nyár.) Španiel, Al-Shehbaz, D.A.German & Marhold, *Saginalibanotica* Rech. f., and *Tripleurospermumsannineum* (Thieb.) Mouterde. For instance, *E.gilgiana* was collected more than five times in different localities (Mouterde 1970). It is hypothesised that, due to its small size, this species may be easily confused with other *Erophila* species. Moreover, its relatively brief flowering period may have led to its oversight by previous collectors. *Odontarrhenalibanotica*, previously named *Alyssumlibanoticum* Nyár. (Španiel et al. 2015), has been collected at least twice by P.H. Davis and Tanios Boutros. The four others have more restricted distribution as they are known from only one locality. Future investigations may lead to the rediscovery of these taxa, as has occurred with other species that went unreported for nearly 70 years due to inaccurate specimen locality data and a lack of targeted fieldwork. Examples include *Chaerophyllumsyriacum* Heldr. & Ehrenb. ex Boiss., recently synonymized with *C.crinitum* Boiss.; *Daphnepontica* L., previously named *D.libanotica* (El Zein and Bou Dagher-Kharrat 2021), *Hieraciumkneissaeum* Mouterde (El Zein et al. 2020), *Jacobaeamouterdei* (Arènes) Greuter & B.Nord. (Tohmé and Tohmé 2009), *Seneciobertramii* Post (Tohmé and Tohmé 2008), *Salviapeyronii* Boiss. ex Post (Tohmé and Tohmé 2011), *Silenecapitellata* Boiss. (Tohmé and Tohmé 2008), *Orobancheastragali* Mouterde (Tohmé and Tohmé 2009), and Noccaearubescenssubsp.culminicola (Mouterde) D.A.German, previously named *Lepidiumculminicola* Mouterde (German 2022).

For the case of Marrubiumglobosumsubsp.libanoticum (Boiss.) P.H.Davis reported from Jordan (Danin and Fragman-Sapir 2016). The available recent data were deemed inadequate as they lacked location, elevation, date, and photographic evidences, and the taxon therefore remained endemic. The new report does not match with the ecology of this oro-Mediterranean species, which occurs above 1,600 meters asl in localities with consequent rainfall (Davis et al. 1972; Mouterde 1984).

Further genetic analyses are recommended to clarify the classification of the nine doubtful taxa (Suppl. material 2). For instance, *Alliumzebdanense* Boiss. & Noe, found on humid and non-calcareous habitats, was initially considered endemic to Mount Lebanon and Anti-Lebanon, but it was later synonymized with *A.chionanthum* Boiss., which is found further in eastern Anatolia in Erzurum on dry limestones (Davis et al. 1984). The status remains uncertain given the considerable disjunction and the notable difference in habitats. Significant similarities between *Corynephorusdeschampsioides* Bornm. and *Corynephorusarticulatus* (Desf.) P.Beauv. lead to the proposition of merging the two taxa (Mouterde 1966). The status of *Cousiniahermonis* requires genetic analyses, as it was synonymized with *C.dayi* Post in the flora of reference (Mouterde 1984). Moreover, another study based on morphological examination of specimens confirmed the complexity of this genus (Mehregan and Kadereit 2008). In a comprehensive study on the genus *Erysimum* (Yildirimli 2008), *Erysimumverrucosum* Boiss. & Gaill. was reported from eastern Turkey. However, the specimens and photos do not match the individuals collected at Mount Hermon, except for the presence of verrucose siliques. Considering that *Erysimum* is a complex genus and that *E.verrucosum* was previously considered as *E.smyrnaeum* Boiss. & Balansa, genetic analyses could help clarify its taxonomic status (Davis et al. 1965; Mouterde 1970). The case of *Erysimumlibanoticum* Post is also complex, as it has been considered a synonym of *E.purpureum* J.Gay (Polatschek 2010), however, the two species are morphologically distinct. Neither the description of the physiognomy nor the ecological characteristics of *E.purpureum* correspond to the type specimen of *E.libanoticum* collected from Makmel. Out of the three specimens used for the genetic analysis, two were collected outside the known distribution range of *E.libanoticum*, from localities at lower elevations, namely the Fort of Hermon, located at 750 m asl, and Yaar (North Baalbek), located at 1,050 m a.sl., likely a misspelling of Yaat. There is a high probability that the specimens sampled were misidentified as *E.libanoticum* but are, in fact, *E.purpureum*, as the latter is commonly found in these areas. Another taxonomic database synonymised *E.libanoticum* with *E.oleifolium* J.Gay (POWO 2025), a species distributed across the steppes extending from Syria to Iran. *Limoniumcedrorum* Domina & Raimondo was described (Domina and Raimondo 2013) based on one specimen collected by Edgecombe in 1966. The locality mentioned on the specimen is the Cedars of Bcharre. However, we suspect a labelling mistake by the collector considering that the presence of this genus in high elevation areas in Lebanon is uncommon. Genetic analyses could reveal any relationship with the usual coastal species of *Limonium*. *Linumtoxicum* Boiss. was historically recorded from a single locality - the summit of Mount Hermon - by six different collectors, including Mouterde. However, Mouterde himself later questioned its taxonomic validity, noting that the distinction between *L.mucronatum* and other subspecies within this group remained unclear (Mouterde 1970). *Ranunculuschionophilus* Boiss. is considered as closely related to *Ranunculuspeyronii* Briq. and the latter was even proposed as subspecies R.chionophilussubsp.sericeus (Peyron) Mouterde (Mouterde 1970). Finally, the exclusion of 48 taxa previously considered as endemic to Lebanon (Suppl. material 3) is the result of a better knowledge on species distribution in the Eastern Mediterranean. For example, *Silenedamascena* Boiss. & Gaill. is historically described as a species strictly endemic to the mountainous areas of Mount Lebanon and Mount Hermon (Boissier 1859), although the name was incorrectly assigned when it was described from Khirbet el-Kneisse in Rashaya District, located far from Damascus. Recently, it was reported from across Golan, Galillee and the West Bank (Danin and Fragman-Sapir 2016).

The endemic taxa are predominantly spermatophytes, except for one species of lycophytes. Considering its position within one of the most ancient extant lineages of vascular plants, the genus *Isoetes* has a relatively high degree of taxonomic diversity and endemism (Brunton and Troia 2018; Troia and Rouhan 2018).

Comparison with the endemic flora of Egypt, Iran and Turkey allowed the characterization of the Lebanese flora. The profile of Lebanon resembles that of Mediterranean and Irano-Turanian regions, where specific families of plants contain the largest numbers of endemic taxa. Among these, Asteraceae holds the highest number of both endemic and non-endemic taxa (Hobohm 2014), while Lamiaceae has a high endemism level in Southwest Asia. Fabaceae, the third largest family in terms of number of species (Xu and Deng 2017), is also one of the most endemic-rich families globally (Hobohm 2014). Additionally, Caryophyllaceae has a center of diversity in the Mediterranean and Irano-Turanian regions (Trigas et al. 2007). The predominance of *Astragalus* can be explained by the fact that its primary center of endemism in the Old-World is located in Southwest Asia (Maassoumi and Ashouri 2022). Similarly, *Allium* has several centers of diversity in the seasonally dry regions of the Northern Hemisphere, including southwestern Asia (Nguyen et al. 2008). Likewise, the diversity within the genus *Centaurea* is the highest in the Irano-Turanian region, with a secondary concentration in the Mediterranean (Wagenitz 1986).

The higher proportions of endemic taxa in the genera *Euphorbia*, *Iris*, *Romulea*, and *Stachys* are notable in Lebanon as they are low or absent in the floras of Egypt, Turkey and Iran. Likewise, Lebanon hosts the richest concentration of *Euphorbia* taxa in Southwest Asia, including non-endemic taxa (Pahlevani et al. 2020). Similarly, the high level of endemism in the Lebanese flora for the Oncocylus section within the Iris genus constitutes one of the main specificities of Levantine floras (Volis et al. 2024). Additionally, the genus *Romulea*, which has centers of diversity in sub-Saharan Africa, Socotra, and the Arabian Peninsula, is not represented by any endemic taxa in Egypt, Iran, and Turkey (Raycheva et al. 2021). Some genera, while not ranked among the twelve most endemic-rich genera in Iran and Turkey, have centers of diversity in the region. This is the case of the genus *Alkanna*, which highest level of endemism is found in the eastern Mediterranean (Chacón et al. 2019). Other genera, such as *Stachys* (Salmaki et al. 2012; Pashova et al. 2024) and *Salvia* (Celep and Doğan 2023), have some of their centers of diversity between the Mediterranean and Western Asia. The comparison with Egypt suggests taxonomic divergences between the Lebanese and the Saharo-Arabian floras, further accentuated by the absence of centers of endemism in the flora of Egypt (Abo Hatab et al. 2024). Despite being the second largest of the four countries, with an area of 1 million km^2^, 100 times larger than Lebanon, Egypt has the lowest number of endemic taxa.

Life forms reflect the strategies adopted by plant species to adapt and survive (Raunkiær 1934). In countries with heterogenous landscapes, such as Turkey and Iran, life forms vary significantly from one region to another (Noroozi et al. 2019a). In contrast, countries with broader geomorphological homogeneity, such as Egypt, or those with limited areas, such as Lebanon, offer a more simplified interpretation of the life forms’ patterns. The predominance of hemicryptophytes in the endemic flora of Lebanon reveals the pronounced mountainous landscape. This adaptation is considered one of the most effective responses to mountainous climatic conditions, and has been observed in the Taurus ranges of Turkey (Parolly 2015), as well as in other mountainous Mediterranean regions (Giménez et al. 2004; Georghiou and Delipetrou 2010; Fois et al. 2022) and in Iran (Noroozi et al. 2019a). In Lebanon, endemic geophytes are represented by only six families in Lebanon, namely Amaryllidaceae, Asparagaceae, Iridaceae, Liliaceae, Orchidaceae and Primulaceae. The bulbous life form is a characteristic trait of the Mediterranean climate and is interpreted as an adaptive strategy to cope with annual droughts in regions with marked seasonality, rocky habitats, and grazing (Blondel 2010). These conditions are common in Lebanon and might have influenced the proportion of endemic geophytes. The low-shrub traits of chamaephytes are more complex to interpret, as they are prevalent in various environmental conditions, including high salinity, strong winds, extreme temperatures, and heavy grazing (Cain 1950). The three taxa from the Mediterranean coast of Lebanon are chamaephytes, while the others are distributed across contrasting mountainous conditions similar to those found in Iran (Noroozi et al. 2019a) and Turkey (Parolly 2015). In the low-altitude regions of the Mediterranean, particularly in the eastern half, therophytes dominate the flora (Quézel 1995). This predominance is also characteristic of regions broadly included within the Saharo-Arabian floristic region (Noroozi et al. 2019a; El-Khalafy et al. 2021b, 2021a; Makhlouf et al. 2023). In both cases, it can be interpreted as an adaptation to prolonged seasonally marked drought or even aridity, as well as to habitats with changing conditions, including anthropic disturbances (Blondel 2010). Apart from Mount Lebanon, that receives significant rainfall, the other half of the country, especially North Beqaa and Anti-Lebanon range, are semi-arid regions. Contrary to expectations, the number of endemic therophytes in these regions was lower than anticipated. Finally, the low representation of endemic phanerophytes, represented by only three families, Berberidaceae, Fagaceae, and Rosaceae, can be primarily explained by their relatively small number of taxa (around 120) in the flora.

The high rate of endemism in Mount Lebanon (70) can be attributed to its unique geographic position, relatively high elevation, and coastal alignment along the Mediterranean Sea. Its summits, reaching up to 3,000 meters, have influenced the formation of diverse contrasting habitats. The presence of coniferous forests of *Abiescilicica* (Antoine & Kotschy) Carrière with *Cedruslibani* A.Rich. on the western slopes of Mount Lebanon (Beals 1965; Talhouk et al. 2001) reflects markedly different environmental conditions compared to the predominantly more arid eastern slopes (Gintzburger et al. 2006). In contrast; Anti-Lebanon harbors a lower concentration of endemic taxa (21), probably due to less pronounced environmental contrasts between its western and eastern slopes (Reifenberg 1952). The topographic isolation of organisms in mountains is also considered a key factor contributing to the higher endemism rate (Thompson et al. 2005; Steinbauer et al. 2016). The absence of suitable habitats for species migration reduces gene flow and increases speciation rates in mountainous areas, similar to the processes observed on islands (Hobohm 2014). This phenomenon is evident in Mount Lebanon, Mount Hermon, and Anti-Lebanon, which constitute respectively the first, second and third highest peaks in the Levant. Spatially isolated, the most similar mountainous habitats are found in the Taurus ranges in Turkey.

The Beqaa has no strictly endemic species, probably due to its narrow width. The taxa found there are typically associated with one or both flanks of the mountain ranges. Moreover, the extensive level of cultivation in the Beqaa leaves little space for wild vegetation (Blondel 2010). Except for the North Beqaa, which has a semi-arid vegetation similar to that of the Anti-Lebanon range, most other areas of Beqaa are heavily exploited for agricultural production (Dal et al. 2021). In South Lebanon, no taxa are strictly endemic. Instead, the flora includes taxa with wider distributions that extend into the Galilee and further south, such as *Irislortetti* Barbey. and *I.bismarckiana* Regel (Gedeon and Khalilieh 2024). The Homs Gap and the northern region of Mount Lebanon, more precisely the Akkar Governorate, share four endemic taxa, *Irisbasaltica* Dinsm., *Isoeteslibanotica* Musselman, Bolin & R. D. Bray, *Lathyrusbasalticus* Rech. f., and *Viciahyaeniscyamus* Mouterde, which are restricted to basaltic substrates. Taxa exclusive to the Mediterranean coast of Lebanon include *Matthiolacrassifolia* Boiss. & Gaill., *Limoniummouterdei* Domina, Erben & Raimondo, and *L.postii* Domina, Erben & Raimondo. Other coastal taxa have wider distributions extending throughout the Levantine seashore, such as *Astragalusberytheus* Boiss. & C.I.Blanche, or *Crepisaculeata* (DC.) Boiss. Mount Hermon hosts only two exclusive endemic taxa but shows greater floristic similarities with Mount Lebanon than with Anti-Lebanon. Nevertheless, all three mountain ranges share a relatively important number of endemic taxa, indicating their close proximity.

## ﻿Conclusion

This study presents the first comprehensive list of plants endemic to Lebanon (169 taxa). It consolidates fragmented information from various publications and verifies herbarium specimens. Endemism patterns are analyzed through taxonomic profile, highlighting the most endemic-rich families and genera, and comparing them with the endemic floras of Turkey, Iran, and Egypt, respectively belonging to the Mediterranean, Irano-Turanian and Saharo-Arabian floristic regions. Family-level comparisons reveal the predominance of Asteraceae, Lamiaceae and Fabaceae, which are usually among the most endemic-rich plant families in the Mediterranean. In contrast, genus-level comparisons offer detailed insights into the Levantine character of the flora, located at biogeographic intersection. This includes the prevalence of Irano-Turanian genera, such as *Astragalus*, *Centaurea*, *Allium*, and *Cousinia*, alongside eastern Mediterranean genera, well represented in Turkey, namely *Galium*, *Salvia*, *Stachys*, *Campanula* and *Alkanna*. Genera such as *Iris*, *Romulea* and *Euphorbia*, with higher endemism proportions, further characterize the floristic distinctiveness of Lebanon. Comparisons with Egypt show minimal similarities, suggesting limited connections to the Saharo-Arabian flora, which is also attributed to the relatively low presence of endemism centers in this region. The mountainous nature of Lebanon is reflected in its high level of endemism and its life form spectrum dominated by hemicryptophytes. The two mountain ranges of Mount Lebanon and Anti-Lebanon emerged as centers of endemism, attributed to their high habitat heterogeneity and topographic isolation.

Ongoing taxonomic changes require a dynamic checklist. Advances in genetics and the increase in botanical observations in neighboring countries, particularly Turkey and Syria, may lead to the extension of distribution ranges for additional taxa. A deeper knowledge of the endemic species in biodiversity hotspots is critical for the effective evaluation and protection of natural heritage. Stakeholders, conservationists and civil society need several tools to estimate the fragility of our environment and natural resources. These plant species embody the local identity of the Levantine region and should be celebrated and preserved. Over half of the taxa have an assessed conservation status, with a high percentage classified as threatened. Therefore, strengthening in situ conservation actions are encouraged, alongside ex situ initiatives for highly threatened taxa.

Endemic taxa in Lebanon, due to their restricted distribution range, mostly qualify as threatened under the criterion of limited geographic range (B), with reduced extent of occupancy and area of occurrence, in conjunction with important degradation of their habitat quality. Extensive monitoring of the populations is required to identify the most effective conservation measures for protecting these plants and their habitats. Additional efforts are needed to complete a larger amount of assessment and improve the general conservation evaluation at the national and regional scales. More than 50% of the endemic species already have an IUCN status at the global level, which allows to seize the risk of extinction and realize the emergency to protect them. Based on the collected data and analyses, the most significant threats to plant and animal diversity have been identified. These include unregulated urban sprawl, and associated road construction, illegal quarries, logging, forest fires, climate change, and overgrazing (El Zein and Kahale 2022). It was also demonstrated that most of the critically and endangered taxa are not located within protected areas, despite the comprehensive mapping of IPAs and KBAs, which has helped identify the priority areas for conservation (Bou Dagher-Kharrat et al. 2018; El Zein et al. 2018). The proportion of threatened species, including taxa classified as CR, EN and VU status, accounts for 47.3% of the species on the list, and 89.9% of the assessed species, highlighting an alarming situation. Urgent actions is needed to designate protected areas, and allocate of public funds, along with staff training for effective management, to ensure the protection of threatened species and their associated natural habitats.

## ﻿Additional information

### Use of AI

No use of AI was reported.

### Author ORCIDs

Hicham El Zein https://orcid.org/0000-0001-7541-9509

Mauro Fois https://orcid.org/0000-0002-4178-0790

Benedetta Gori https://orcid.org/0000-0003-0934-3029

Gianluigi Bacchetta https://orcid.org/0000-0002-1714-3978

### Data availability

The dynamic checklist of plant taxa endemic to Lebanon, including species and subspecies levels, is available online on the Global Biodiversity Information Facility (GBIF) and will be updated as modifications occur (https://doi.org/10.15468/qcnfqb).

## Supplementary materials

Supplementary material 1 Included endemic taxa Data type docx Explanation note List of included endemic plant taxa in Lebanon. https://binary.pensoft.net/file/1383445This dataset is made available under the Open Database License (http://opendatacommons.org/licenses/odbl/1.0/). The Open Database License (ODbL) is a license agreement intended to allow users to freely share, modify, and use this Dataset while maintaining this same freedom for others, provided that the original source and author(s) are credited.Hicham El Zein, Mauro Fois, Benedetta Gori, Gianluigi Bacchetta

Supplementary material 2 Doubtful taxa Data type xlsx Explanation note List of doubtful plant taxa in Lebanon. https://binary.pensoft.net/file/1383446This dataset is made available under the Open Database License (http://opendatacommons.org/licenses/odbl/1.0/). The Open Database License (ODbL) is a license agreement intended to allow users to freely share, modify, and use this Dataset while maintaining this same freedom for others, provided that the original source and author(s) are credited.Hicham El Zein, Mauro Fois, Benedetta Gori, Gianluigi Bacchetta

Supplementary material 3 Excluded taxa Data type xlsx Explanation note List of excluded plant taxa not considered anymore endemic to Lebanon. https://binary.pensoft.net/file/1383447This dataset is made available under the Open Database License (http://opendatacommons.org/licenses/odbl/1.0/). The Open Database License (ODbL) is a license agreement intended to allow users to freely share, modify, and use this Dataset while maintaining this same freedom for others, provided that the original source and author(s) are credited.Hicham El Zein, Mauro Fois, Benedetta Gori, Gianluigi Bacchetta
