# Supplementary material for: ﻿Endemism patterns of the vascular flora of Lebanon: A dynamic checklist
Source: PhytoKeys. 2025 Jul 28;260:153–84. doi: 10.3897/phytokeys.260.156938 (PMC12322683; doi:10.3897/phytokeys.260.156938)
Supplement: Supplementary material 1 — Included endemic taxa [file phytokeys-260-153_article-156938__-s001.docx]

Supplementary file 1. Checklist of vascular plants endemic to Lebanon comprising 169 taxa at the date of publication, with families, basionyms, life forms: Ch= Chamaephyte, G=Geophytes, H=Hemicryptophyte, P=Phanerophyte, T=Therophyte; countries: Leb=Lebanon, Syr=Syria; region in Lebanon: LB=Mount Lebanon, AN=Anti-Lebanon, HE=Mount Hermon, HO=Homs Gap, MC=Mediterranean Coast; IUCN status: CR=Critically endangered, EN=endangered, VU=vulnerable, NT= Near threatened, LC=Least Concern, na=not assessed.

| # | **Accepted name** | **Family** | **Basionym** | **Life form** | **Countries** | **Region** | **IUCN status** |
| --- | --- | --- | --- | --- | --- | --- | --- |
| 1 | *Acantholimon antilibanoticum* Mouterde | Plumbaginaceae |  | Ch | Syr, Leb | LB-AN-HE | na |
| 2 | *Ajuga chasmophila* P.H.Davis | Lamiaceae |  | H | Syr, Leb | AN | na |
| 3 | *Alchemilla diademata* Rothm. | Rosaceae |  | H | Leb | LB | CR |
| 4 | *Alkanna leiocarpa* Rech. f. | Boraginaceae |  | H | Leb | LB | EN |
| 5 | *Alkanna maleolens* Bornm. | Boraginaceae |  | H | Leb | LB | CR |
| 6 | *Alkanna prasinophylla* Rech. f. | Boraginaceae |  | H | Leb | LB | EN |
| 7 | *Allium feinbergii* Oppenheimer | Amaryllidaceae |  | G | Syr, Leb | LB-HE | LC |
| 8 | *Allium hermoneum* (Kollmann & Shmida) Brullo, Guglielmo, Pavone & Salmeri | Amaryllidaceae | *Allium albotunicatum* subsp. *hermoneum* Kollmann & Shmida | G | Syr, Leb | HE | LC |
| 9 | *Allium libani* Boiss*.* | Amaryllidaceae |  | G | Syr, Leb | LB-AN-HE | NT |
| 10 | *Allium machmelianum* Post | Amaryllidaceae | *Allium makmelianum* Post | G | Syr, Leb | LB-AN | NT |
| 11 | *Allium pseudocalyptratum* Mouterde | Amaryllidaceae |  | G | Leb | LB-HE | EN |
| 12 | *Allium pseudostamineum* Kollmann & Shmida | Amaryllidaceae |  | G | Leb | LB-AN-HE | LC |
| 13 | *Allium sannineum* Gombault | Amaryllidaceae |  | G | Leb | LB-HE | EN |
| 14 | *Anthemis didymaea* Mouterde | Asteraceae |  | H | Leb | LB | CR |
| 15 | *Arenaria libanotica* Kotschy | Caryophyllaceae |  | H | Leb | LB-HE | EN |
| 16 | *Astragalus argyrothamnos* Boiss. | Fabaceae |  | Ch | Syr | AN | na |
| 17 | *Astragalus cedreti* Boiss*.* | Fabaceae |  | H | Leb | LB | EN |
| 18 | *Astragalus coluteoides* Will | Fabaceae |  | Ch | Syr, Leb | LB-AN-HE | na |
| 19 | *Astragalus cruentiflorus* Boiss. | Fabaceae |  | Ch | Syr, Leb | LB-AN-HE | na |
| 20 | *Astragalus ehdenensis* Mouterde | Fabaceae |  | H | Leb | LB | EN |
| 21 | *Astragalus ehrenbergii* Bunge | Fabaceae |  | H | Syr, Leb | LB-AN-HE | na |
| 22 | *Astragalus exiguus* Post | Fabaceae |  | Ch | Syr | AN | na |
| 23 | *Astragalus hermoneus* Boiss. | Fabaceae |  | Ch | Syr, Leb | LB-AN-HE | na |
| 24 | *Astragalus hirsutissimus* DC. | Fabaceae |  | H | Leb | LB-HE | EN |
| 25 | *Astragalus kurnet-es-saudae* Eig. | Fabaceae |  | H | Leb | LB | CR |
| 26 | *Astragalus lanatus* Labill. | Fabaceae |  | H | Leb | LB-HE | EN |
| 27 | *Astragalus nummularius* Lam*.* subsp. *trichopterus* (Boiss.) Thiébaut | Fabaceae | *Astragalus trichopterus* Boiss. | H | Syr, Leb | LB-AN | na |
| 28 | *Astragalus psilodontius* Boiss. | Fabaceae |  | Ch | Syr, Leb | AN | na |
| 29 | *Astragalus siliquosus subsp. stramineus* (Boiss.) Kozik & Podlech | Fabaceae | *Astragalus stramineus* Boiss. | H | Syr | AN | na |
| 30 | *Atocion reuterianum* (Boiss. & C.I.Blanche) Frajman | Caryophyllaceae | *Silene reuteriana* Boiss. & C.I.Blanche | H | Leb | LB | VU |
| 31 | *Atriplex zahlensis* Mouterde | Chenopodiaceae |  | T | Leb | LB | na |
| 32 | *Ballota antilibanotica* Post | Lamiaceae |  | H | Syr, Leb | LB-AN | na |
| 33 | *Ballota byblensis* Semaan & R.M.Haber | Lamiaceae |  | H | Leb | LB | na |
| 34 | *Barbarea macrocarpa* (Boiss. & Heldr.) Al-Shehbaz & Jacquemoud | Brassicaceae |  | H | Leb | LB-HE | EN |
| 35 | *Bellevalia hermonis* Mouterde | Asparagaceae |  | G | Syr, Leb | LB-HE | LC |
| 36 | *Berberis libanotica* Ehrenb*.* | Berberidaceae |  | P | Syr, Leb | LB-AN-HE | LC |
| 37 | *Bupleurum postii* Wolff. | Apiaceae |  | T | Syr, Leb | AN | na |
| 38 | *Campanula antilibanotica* (P.H.Davis) Greuter & Burdet | Campanulaceae | *Tracheliopsis antilibanotica* P.H.Davis | H | Syr, Leb | LB-AN | na |
| 39 | *Campanula damascena* Labill. | Campanulaceae |  | H | Syr, Leb | LB-AN-HE | na |
| 40 | *Campanula euclasta* Boiss. | Campanulaceae |  | H | Syr, Leb | LB-AN | na |
| 41 | *Centaurea ainetensis* Boiss. | Asteraceae |  | H | Syr, Leb | LB-AN | na |
| 42 | *Centaurea drabifolia* Sibth. & Sm. subsp. *libanotica* Boiss. | Asteraceae |  | H | Syr, Leb | LB-AN-HE | na |
| 43 | *Centaurea heterocarpa* Boiss. & Gaill. ex Boiss. | Asteraceae |  | H | Leb | LB-HE | EN |
| 44 | *Centaurea hololeuca* Boiss. | Asteraceae |  | H | Syr, Leb | LB-AN-HE | na |
| 45 | *Centaurea iberica* subsp. *hermonis* (Boiss.) Bornm. | Asteraceae | *Centaurea hermonis* Boiss. | H | Syr, Leb | AN-HE | na |
| 46 | *Centaurea iberica* subsp. *meryonis* (DC.) Bornm. | Asteraceae | *Centaurea meryonis* DC. | H | Syr, Leb | LB | na |
| 47 | *Centaurea longispina* (Post) Wagenitz | Asteraceae |  | H | Syr | AN | na |
| 48 | *Centaurea mouterdei* Wagenitz | Asteraceae |  | H | Leb | LB | EN |
| 49 | *Cephalaria cedrorum* Mouterde | Dipsacaceae |  | H | Leb | LB | VU |
| 50 | *Cephalaria kesruanica* Mouterde | Dipsacaceae |  | H | Leb | LB | EN |
| 51 | *Chaerophyllum aurantiacum* Post | Apiaceae |  | H | Syr, Leb | LB-HE | EN |
| 52 | *Cherleria rupestris* (Labill.) A.J.Moore & Dillenb | Caryophyllaceae | *Minuartia labillardieri* Briquet | H | Leb | LB | EN |
| 53 | *Cirsium leucocephalum* subsp. *hermonis* (Boiss.) Greuter | Asteraceae | *Cirsium hermonis* Boiss. | H | Syr, Leb | LB-AN-HE | VU |
| 54 | *Cistus umbellatus* subsp. *libani* Demoly | Cistaceae |  | Ch | Leb | LB | EN |
| 55 | *Clinopodium libanoticum* (Boiss.) Kuntze | Lamiaceae | *Micromeria libanotica* Boiss. | H | Syr, Leb | LB-AN | EN |
| 56 | *Clinopodium nummulariifolium* (Boiss.) Kuntze | Lamiaceae | *Micromeria nummulariifolia* Boiss. | H | Leb | LB | EN |
| 57 | *Cota lyonnetioides* Boiss. & Kotschy | Asteraceae | *Anthemis lyonnetioides* Boiss*.* | T | Leb | AN-HE | na |
| 58 | *Cousinia libanotica* DC. | Asteraceae |  | H | Leb | LB | VU |
| 59 | *Cousinia pestalozzae* Boiss. | Asteraceae |  | H | Syr, Leb | AN | na |
| 60 | *Crepis robertioides* Boiss. | Asteraceae |  | H | Syr, Leb | LB-AN-HE | EN |
| 61 | *Crocus baalbekensis Addam & Bou-Hamdan* | Iridaceae |  | Geo | Leb | AN | na |
| 62 | *Cyclamen libanoticum* Hildebr. | Primulaceae |  | G | Leb | LB | EN |
| 63 | *Dactylorhiza phoenissa* (B.Baumann & H.Baumann) P.Delforge | Orchidaceae |  | G | Leb | LB | na |
| 64 | *Dianthus karami* Bl. | Caryophyllaceae |  | H | Leb | LB | EN |
| 65 | *Draba antilibanotica* Al-Shehbaz | Brassicaceae | *Draba oxycarpa* Boiss. & Heldr. | H | Syr, Leb | LB-AN-HE | EN |
| 66 | *Draba gilgiana* Muschl. | Brassicaceae |  | T | Leb | LB | na |
| 67 | *Draba vesicaria* Desv. | Brassicaceae |  | H | Syr, Leb | LB-AN-HE | na |
| 68 | *Eleocharis macrantha* Bockel | Cyperaceae |  | H | Leb | LB | LC |
| 69 | *Erigeron libanoticus* Vierh. | Asteraceae |  | H | Leb | LB | EN |
| 70 | *Erodium trichomanifolium* L'Hér. | Geraniaceae |  | H | Syr, Leb | LB-HE | na |
| 71 | *Euphorbia antilibanotica* Mouterde | Euphorbiaceae |  | H | Syia, Leb | AN-HE | na |
| 72 | *Euphorbia caudiculosa* Boiss. | Euphorbiaceae |  | H | Syia, Leb | LB-HE | na |
| 73 | *Euphorbia erinacea* Boiss. & Kotschy. | Euphorbiaceae |  | Ch | Syia, Leb | AN-HE | na |
| 74 | *Euphorbia promecocarpa* Davis | Euphorbiaceae |  | H | Syr, Leb | LB-AN | na |
| 75 | *Ferula hermonis* Boiss. | Apiaceae |  | H | Syr, Leb | AN-HE | EN |
| 76 | *Ferulago westii* (Post) Pimenov & Kljuykov | Apiaceae | *Johrenia westii* Post | H | Leb | LB-AN | EN |
| 77 | *Gagea micrantha* (Boiss.) Pascher | Liliaceae | *Gagea foliosa* var. *micrantha* Boiss. | G | Syr, Leb | LB-AN-HE | VU |
| 78 | *Galium jungermannioides* Boiss. | Rubiaceae |  | H | Leb | LB | na |
| 79 | *Galium libanoticum* Ehrend. | Rubiaceae |  | H | Syr, Leb | LB-AN-HE | na |
| 80 | *Galium pestalozzae* Boiss. | Rubiaceae |  | H | Leb | LB | VU |
| 81 | *Galium thiebautii* Ehrend. | Rubiaceae |  | H | Leb | LB-AN | EN |
| 82 | *Gelasia mackmeliana* (Boiss.) Zaika, Sukhor. & N.Kilian | Asteraceae | *Scorzonera mackmeliana* Boiss. | H | Leb | LB-AN-HE | EN |
| 83 | *Geranium makmelicum* Aedo | Geraniaceae |  | H | Leb | LB | na |
| 84 | *Hedysarum coelesyriacum* Sam. | Fabaceae |  | H | Syr, Leb | LB-AN | na |
| 85 | *Helichrysum pygmaeum* Post | Asteraceae |  | Ch | Syr, Leb | AN | na |
| 86 | *Helichrysum virgineum* DC. | Asteraceae |  | H | Leb | LB | EN |
| 87 | *Heliotropium schweinfurthii* Boiss. | Boraginaceae |  | T | Syr, Leb | AN | na |
| 88 | *Hieracium kneissaeum* Mouterde | Asteraceae |  | H | Leb | LB | CR |
| 89 | *Hieracium schmidtii* subsp. *libanoticum* (Boiss. & C.I.Blanche) Greuter | Asteraceae |  | H | Leb | LB | na |
| 90 | *Hypericum libanoticum* N.Robson | Hypericaceae |  | H | Syr, Leb | LB-AN-HE | VU |
| 91 | *Iris antilibanotica* Dinsm. | Iridaceae |  | G | Syr, Leb | AN | CR |
| 92 | *Iris basaltica* Dinsm. | Iridaceae |  | G | Syr, Leb | LB-HO | na |
| 93 | *Iris cedreti* Dinsm. | Iridaceae |  | G | Leb | LB | CR |
| 94 | *Iris susiana* L. | Iridaceae |  | G | Leb | LB | EN |
| 95 | *Iris westii* Dinsm. | Iridaceae |  | G | Syr, Leb | LB-HE | EN |
| 96 | *Isoetes libanotica* Musselman, Bolin & R.D.Bray | Isoetaceae |  | H | Syr, Leb | LB-HO | na |
| 97 | *Jacobaea mouterdei* (Arènes) Greuter & B.Nord | Asteraceae | *Senecio mouterdei* Arenes | H | Leb | LB | EN |
| 98 | *Klasea mouterdei* (Arènes) Greuter & Wagenitz | Asteraceae | × *Centauserratula mouterdei* Arènes | H | Leb | LB | CR |
| 99 | *Lathyrus basalticus* Rech. f. | Fabaceae |  | T | Syr, Leb | LB-HO | na |
| 100 | *Leontodon libanoticus* Boiss. | Asteraceae |  | H | Leb | LB | VU |
| 101 | *Limonium mouterdei* Domina, Erben & Raimondo | Plumbaginaceae |  | Ch | Leb | MC | na |
| 102 | *Limonium postii* Domina, Erben & Raimondo | Plumbaginaceae |  | Ch | Leb | MC | na |
| 103 | *Linum carnosulum* Boiss. | Linaceae |  | H | Leb | LB | CR |
| 104 | *Lycochloa avenacea* Sam. | Poaceae |  | H | Leb | LB | CR |
| 105 | *Marrubium globosum* subsp. *libanoticum* (Boiss.) P.H.Davis | Lamiaceae | *Marrubium libanoticum* Boiss. | H | Syr, Leb | LB-AN-HE | na |
| 106 | *Matthiola crassifolia* Boiss. & Gaill*.* | Brassicaceae |  | Ch | Leb | MC | EN |
| 107 | *Micromeria graeca* (L.) Benth. subsp. *laxiflora* (Post) Mouterde | Lamiaceae | *Micromeria graeca* var. *laxiflora* Post | H | Leb | LB | na |
| 108 | *Minuartia innominata* McNeill | Caryophyllaceae |  | H | Syr, Leb | AN | na |
| 109 | *Minuartia libanotica* (Boiss.) Bornm. | Caryophyllaceae | *Alsine libanotica* Boiss. | H | Leb | LB | EN |
| 110 | *Minuartia parvulorum* Mouterde & Sam. ex Rech. f. | Caryophyllaceae |  | H | Syr, Leb | AN | na |
| 111 | *Myopordon pulchellum* (Winkler & Barbey) Wagenitz | Asteraceae | *Autrania pulchella* C.Winkl. & Barbey | H | Leb | LB | EN |
| 112 | *Noccaea rubescens* subsp. *culminicola* (Mouterde) D.A.German | Brassicaceae | *Lepidium culminicola* Mouterde | H | Leb | LB | na |
| 113 | *Odontarrhena libanotica* (Nyár.) Španiel, Al-Shehbaz, D.A.German & Marhold | Brassicaceae | *Alyssum libanoticum* Nyár. | H | Leb | LB | DD |
| 114 | *Odontites hispidulus* (Boiss.) Bolliger | Scrophulariaceae | *Odontites luteus* var. *hispidulus* Boiss. | T | Leb | LB | EN |
| 115 | *Onosma sanninensis Maalouf & Binzet* | Boraginaceae |  | Hem | Leb | LB | CR |
| 116 | *Origanum ehrenbergii* Boiss. | Lamiaceae |  | H | Leb | LB | VU |
| 117 | *Origanum libanoticum* Boiss. | Lamiaceae |  | H | Leb | LB | VU |
| 118 | *Ornithogalum libanoticum* Boiss. | Asparagaceae |  | G | Leb | LB | EN |
| 119 | *Orobanche astragali* Mouterde | Orobanchaceae |  | H | Leb | LB | EN |
| 120 | *Papaver libanoticum* Boiss. subsp. *libanoticum* | Papaveraceae |  | H | Syr, Leb | LB-AN-HE | EN |
| 121 | *Phagnalon linifolium* Post | Asteraceae |  | H | Syr, Leb | AN | na |
| 122 | *Phlomis brevilabris* Ehrenb. | Lamiaceae |  | H | Syr, Leb | LB-AN-HE | na |
| 123 | *Phlomis tathamiorum* R.M.Haber & Semaan | Lamiaceae |  | H | Leb | LB | na |
| 124 | *Polygonum libani* Boiss. | Polygonaceae |  | H | Syr, Leb | LB-AN-HE | na |
| 125 | *Prangos asperula* Boiss. subsp. *asperula* | Apiaceae |  | H | Syr, Leb | LB-AN | na |
| 126 | *Prunus boissieri* Kurtto | Rosaceae | *Amygdalus agrestis* Boiss. | P | Leb | AN | EN |
| 127 | *Quercus kotschyana* O.Schwarz | Fagaceae |  | P | Leb | LB | EN |
| 128 | *Ranunculus orbiculatus* Blanche | Ranunculaceae |  | H | Leb | LB | EN |
| 129 | *Ranunculus schweinfurthii* Boiss. | Ranunculaceae |  | H | Leb | LB | EN |
| 130 | *Rhanteriopsis lanuginosa* (DC.) Rauschert | Asteraceae | *Asteriscus lanuginosus* Aucher ex DC. | H | Syr, Leb | LB-AN | na |
| 131 | *Rhanteriopsis microcephala* (Boiss.) Rauschert | Asteraceae | *Postia microcephala* Boiss. | T | Syr, Leb | AN | na |
| 132 | *Romulea jezzinis* K.Addam & M.Bou-Hamdan | Iridaceae |  | G | Leb | LB | na |
| 133 | *Romulea libanotica* K.Addam & M.Bou-Hamdan | Iridaceae |  | G | Leb | LB | na |
| 134 | *Romulea nivalis* (Boiss. & Ky) Klatt | Iridaceae | *Trichonema nivale* Boiss. & Kotschy | G | Syr, Leb | LB-AN-HE | na |
| 135 | *Romulea phoenicia*Mouterde | Iridaceae |  | G | Leb | LB | VU |
| 136 | *Rosa schergiana* Boiss. | Rosaceae |  | P | Syr, Leb | AN | na |
| 137 | *Rumex angustifolius* Campd. subsp. *libanoticus* Rech. f. | Polygonaceae |  | H | Leb | LB | na |
| 138 | *Sagina libanotica* Rech. f. | Caryophyllaceae |  | H | Leb | LB | CR |
| 139 | *Salvia fairuziana* R.M.Haber & Semaan | Lamiaceae |  | H | Leb | LB | na |
| 140 | *Salvia josetta* El Zein | Lamiaceae |  | H | Leb | LB | CR |
| 141 | *Salvia peyronii* Boiss. ex Post | Lamiaceae |  | H | Leb | LB | CR |
| 142 | *Salvia rubifolia* Boiss. | Lamiaceae |  | H | Syr, Leb | LB-AN-HE | na |
| 143 | *Scilla libanotica* Speta | Asparagaceae |  | G | Syr, Leb | LB-AN-HE | LC |
| 144 | *Scorzonera libanotica* Boiss. | Asteraceae |  | H | Syr, Leb | LB-AN-HE | EN |
| 145 | *Scutellaria utriculata* Labill. | Lamiaceae |  | H | Syr, Leb | LB-AN-HE | na |
| 146 | *Senecio bertramii* Post | Asteraceae |  | H | Leb | LB | CR |
| 147 | *Senecio blanchei* Soldano | Asteraceae | *Senecio exilis* C.I.Blanche ex Boiss. | T | Leb | LB | CR |
| 148 | *Silene astartes* C.I.Blanche | Caryophyllaceae |  | H | Leb, Syr | LB-HE | VU |
| 149 | *Silene libanotica* Boiss. | Caryophyllaceae |  | H | Syr, Leb | LB-AN-HE | na |
| 150 | *Stachys ehrenbergii* Boiss. | Lamiaceae |  | H | Syr, Leb | LB-HE | VU |
| 151 | *Stachys hydrophila* Boiss. | Lamiaceae |  | H | Leb | LB | VU |
| 152 | *Stachys nivea* Labill. | Lamiaceae |  | Ch | Syr, Leb | LB-AN-HE | na |
| 153 | *Stachys paneiana* Mouterde | Lamiaceae |  | H | Syr, Leb | HE | na |
| 154 | *Sterigmostemum billardierei* (DC.) D.A.German | Brassicaceae |  | H | Syr, Leb | LB-AN-HE | VU |
| 155 | *Tanacetum densum* subsp. *densum* (Labill.) Sch. Bip. | Asteraceae | *Pyrethrum densum* Labill. | Ch | Syr, Leb | AN-HE | na |
| 156 | *Teucrium antilibanoticum* Mouterde | Lamiaceae |  | H | Syr, Leb | AN | na |
| 157 | *Teucrium montbretii* Benth. subsp. *libanoticum* Davis | Lamiaceae |  | H | Leb | LB | VU |
| 158 | *Teucrium socinianum* Boiss. | Lamiaceae |  | Ch | Syr, Leb | LB-AN | na |
| 159 | *Thesium libanoticum* Ehrenb. | Santalaceae |  | H | Leb | LB | EN |
| 160 | *Thymus alfredae* Post | Lamiaceae |  | Ch | Syr, Leb | AN | na |
| 161 | *Trifolium meduseum* C.I.Blanche ex Boiss. | Fabaceae |  | H | Syr, Leb | LB-AN-HE | na |
| 162 | *Trifolium sannineum* Mouterde | Fabaceae |  | T | Leb | LB | EN |
| 163 | *Tripleurospermum sannineum* (J.Thiébaut) P.Mouterde ex Charpin & Dittrich | Asteraceae | *Chamaemelum sannineum* J.Thiébaut | H | Leb | LB | CR |
| 164 | *Valerianella antilibanotica* Rech.f. | Caprifoliaceae |  | T | Syr, Leb | AN | na |
| 165 | *Verbascum antilibanoticum* Hub.-Mor | Scrophulariaceae |  | H | Syr, Leb | LB-AN | na |
| 166 | *Veronica bombycina* subsp. *bombycina* Boiss. & Kotschy | Scrophulariaceae |  | H | Syr, Leb | LB-AN | na |
| 167 | *Veronica caespitosa* subsp. *leiophylla* (Boiss.) M.A.Fisch. | Scrophulariaceae |  | H | Leb | LB | EN |
| 168 | *Vicia hyaeniscyamus* Mouterde | Fabaceae |  | T | Syr, Leb | LB-HO | na |
| 169 | *Viola libanotica* Boiss. | Violaceae |  | H | Leb | LB | EN |

Supplementary file 2. List of plant taxa with doubtful taxonomic status, previously considered endemic to Lebanon, and for which further investigations are required. References and explanations about their uncertain status are given.

| # | **Name in reference flora** |  | **Family** | **Explanations** | **References** |
| --- | --- | --- | --- | --- | --- |
| 1 | *Allium zebdanense* Boiss. & Noe |  | Amaryllidaceae | Synonymized with *A*. *chionanthum* | Davis *et al.* 1984 |
| 2 | *Astragalus hispidus* Labill. |  | Fabaceae | Synonymized with *A*. *nanus* from eastern Turkey and then considered again endemic to Lebanon. | Podlech & Zarre 2013 |
| 3 | *Corynephorus deschampsioides* Bornm. |  | Poaceae | Probably a variety of *C. articulatus* according to Mouterde | Mouterde 1966 |
| 4 | *Cousinia hermonis* Boiss. |  | Asteraceae | Similarities with *Cousinia dayi* Post. | Mehregan and Kadereit 2008 |
| 5 | *Erysimum libanoticum* Post |  | Brassicaceae | Synonymized with *E. oleifolium* J.Gay on POWO or with *E. purpureum* J.Gay by Polatschek. | Polatschek 2010;  POWO 2025 |
| 6 | *Erysimum verrucosum* Boiss. & Gaill. |  | Brassicaceae | Reported from eastern Turkey. Large disjunct distribution | Yildirimli 2008 |
| 7 | *Linum toxicum* Boiss. |  | Linaceae | Very similar to *L. mucronatum* Bertol according to Mouterde | Mouterde 1970 |
| 8 | *Ranunculus chionophilus* Boiss. |  | Ranunculaceae | Closely related to *R. peyronii* Briq. according to Mouterde | Mouterde 1970 |
| 9 | *Trifolium farayense* Mouterde |  | Fabaceae | Probably the same taxon as *T. sannineum* according to Mouterde. | Mouterde 1970 |

References

Davis PH, Mill RR, Tan K (1984) 8 Flora of Turkey and the East Aegean Islands, Volume 8. Edinburgh University Press, Edinburgh, UK, 632 pp.

Mehregan I, Kadereit JW (2008) Taxonomic Revision of Cousinia sect. Cynaroideae (Asteraceae, Cardueae). Willdenowia 38: 293–362.

Mouterde P (1966) 1 Nouvelle flore du Liban et de la Syrie. Dar El-Machreq, Beirut, Lebanon, 642 pp.

Mouterde P (1970) 2 Nouvelle flore du Liban et de la Syrie. Dar El-Machreq, Beirut, Lebanon, 720 pp.

Podlech D, Zarre S (2013) A Taxonomic Revision of the Genus Astragalus L. (Leguminosae) in the Old World. Naturhistorisches Museum Wien, Vienna, Austria, 2439 pp.

Polatschek A (2010) Revision der Gattung Erysimum (Cruciferae), Teil 2: Georgien, Armenien, Azerbaidzan, Türkei, Syrien, Libanon, Israel, Jordanien, Irak, Iran, Afghanistan. Annalen des Naturhistorischen Museums in Wien. Serie B, Fur Botanik und Zoologie 112: 369–497.

POWO (2025) Plants of the World Online. Plants of the World Online. Available from: http://www.plantsoftheworldonline.org/ (January 25, 2025).

Yildirimli Ş (2008) The genus Erysimum L. (Brassicaceae) in Turkey, some new taxa, records, a synopsis and a key. Ot Sistematik Botanik Dergisi 15: 1–80.

Supplementary file 3. List of taxa previously considered endemic to Lebanon, but that were found to have wider distributions, ordered here by the names used in Flora of Syria and Lebanon (Mouterde 1966, 1970, 1984), along with their family, accepted names (POWO 2025), basionym, countries of occurrence: Afg=Afghanistan, Bul=Bulgaria, Cas=Central Asia (Kazakhstan, Kyrgyzstan, Tajikistan, Turkmenistan, Uzbekistan), Cau=Caucasus (Armenia, Azerbaijan, Georgia), Cyp=Cyprus, Eg=Egypt, Gr=Greece, Irn=Iran, Irq=Iraq, Jo=Jordan, Lb=Lebanon, Lib=Libya, Med=all Mediterranean countries, Pal=Palestine/Israel, Sau=Saudi Arabia, Syr=Syria, Tur=Turkey, IUCN global status, the bibliographic references and the reason of their exclusion.

| # | **Name Mouterde** | **Family** | **Accepted name** | **Basionym** | **Countries** | **National IUCN status** | **References** | **Reason** |
| --- | --- | --- | --- | --- | --- | --- | --- | --- |
| 1 | *Acantholimon libanoticum* Boiss*.* | Plumbaginaceae |  |  | Tur, Syr, Lb, Pal |  | Davis et al. 1982 | Recorded elsewhere |
| 2 | *Aethionema oppositifolium* (Labill.) Boiss. | Brassicaceae | *Noccaea oppositifolia* (Pers.) Al-Shehbaz & Menke | *Iberis oppositifolia* Pers. | Tur, Lb | EN | Davis et al. 1965 | Recorded elsewhere |
| 3 | *Aethionema stylosum* DC. | Brassicaceae |  |  | Tur, Lb |  | Davis et al. 1965 | Recorded elsewhere |
| 4 | *Agropyron libanoticum* Hack. | Poaceae | *Pseudoroegneria libanotica* (Hack.) D.R.Dewey |  | Tur, Syr, Lb, Pal |  | Davis et al. 1985 | Recorded elsewhere |
| 5 | *Allium rupicolum* Boiss. | Amaryllidaceae | *Allium rupicola* Boiss. ex Mouterde |  | Gr, Tur, Lb |  | Davis et al. 1984 | Recorded elsewhere |
| 6 | *Althaea bertramii* Post & Beauv. | Malvaceae |  |  |  |  | CHG 2025 G00353161 https://www.ville-ge.ch/musinfo/bd/cjb/chg/adetail.php?id=249525&lang=en | Misdescription |
| 7 | *Alyssum condensatum* Boiss. & Hausskn. subsp*. flexibile* (Nyaradi) Dudley | Brassicaceae | *Odontarrhena condensata subsp. flexibilis* (Nyar.) Spaniel & al. | *Alyssum flexibile* Nyár. | Tur, Syr, Lb, Irq |  | Davis et al. 1965 | Recorded elsewhere |
| 8 | *Asperula libanotica* Boiss. | Rubiaceae |  |  | Lb, Pal |  | Danin & Fragman-Sapir 2025 | Recorded elsewhere |
| 9 | *Asphodeline damascena* Boiss. subsp. *damascena* | Liliaceae |  |  | Tur, Syr, Lb | LC | Davis et al. 1984 | Recorded elsewhere |
| 10 | *Astragalus angulosus* DC. | Fabaceae |  |  | Syr, Lb, Pal, Jo |  | Podlech & Zarre 2013 | Taxonomic revision |
| 11 | *Astragalus baalbekensis* Bornm. | Fabaceae | *Astragalus bethlehemiticus* Boiss. |  | Tur, Syr, Lb, Pal, Jo |  | Podlech & Zarre 2013 | Taxonomic revision |
| 12 | *Astragalus dictyocarpus* Boiss. | Fabaceae | *Astragalus angulosus* DC. |  | Syr, Lb, Pal, Jo |  | Podlech & Zarre 2013 | Taxonomic revision |
| 13 | *Astragalus echinus* DC. | Fabaceae | *Astragalus echinus* subsp. *echinus* DC. | *Astragalus libanoticus* Boiss. | Syr, Lb, Eg, Jo, Sau |  | Podlech & Zarre 2013 | Recorded elsewhere |
| 14 | *Astragalus emarginatus* Labill. | Fabaceae |  |  | Tur, Syr, Lb, Irq |  | Davis et al. 1970 | Recorded elsewhere |
| 15 | *Astragalus gaillardotii* Boiss. | Fabaceae | *Astragalus oleifolius* DC. |  | Tur, Syr, Lb, Irq, Irn, Cau |  | Podlech & Zarre 2013 | Taxonomic revision |
| 16 | *Astragalus pabotii* Mouterde | Fabaceae | *Astragalus kerkukensis* Bornm. |  | Syr, Irq, Irn, Afg |  | Podlech & Zarre 2013 | Taxonomic revision |
| 17 | *Astragalus sofarensis* Thiebaut | Fabaceae | *Astragalus oleifolius* DC. |  | Tur, Syr, Lb, Irq, Irn, Cau |  | Podlech & Zarre 2013 | Taxonomic revision |
| 18 | *Astragalus trifoliolatus* Boiss. | Fabaceae |  |  | Syr, Lb, Irq, Irn |  | Podlech & Zarre 2013 | Recorded elsewhere |
| 19 | *Centaurea damascena* Boiss. | Asteraceae |  |  | Lb, Syr |  | Danin 2023 | Recorded elsewhere |
| 20 | *Chaerophyllum syriacum* Hempr. & Ehrenb. | Apiaceae | *Chaerophyllum crinitum* Boiss. |  | Tur, Lb, Irq, Irn, Cau | CR | Piwczyński *et al.* 2015 | Taxonomic revision |
| 21 | *Convolvulus libanoticus* Boiss. | Convolvulaceae |  |  | Tur, Syr, Lb, Pal, Cre |  | Davis et al. 1978 | Recorded elsewhere |
| 22 | *Cytisus syriacus* Boiss. & Bl. | Fabaceae | *Genista monspessulana* (L.) L.A.S.Johnson | *Genista syriaca* Boiss. & C.I.Blanche | Med, except Lib, Eg, Pal | EN | POWO 2025 | Taxonomic revision |
| 23 | *Dianthus pendulus* Boiss. & Bl. | Caryophyllaceae |  |  | Lb, Pal, Jo, Irq, Irn |  | Safikhani & Mahmoodi 2020 | Taxonomic revision |
| 24 | *Erodium gaillardotii* Boiss. | Geraniaceae |  |  | Tur, Syr, Lb |  | Davis et al. 1967 | Recorded elsewhere |
| 25 | *Ferulago frigida* Boiss. | Apiaceae | *Ferulago trachycarpa* Boiss*.* |  | Tur, Syr, Lb |  | Bernardi 1979 | Taxonomic revision |
| 26 | *Genista libanotica* Boiss. | Fabaceae |  |  | Tur, Syr, Lb |  | Davis et al. 1970 | Recorded elsewhere |
| 27 | *Geranium libanoticum* Schenk | Geraniaceae |  |  | Tur, Syr, Lb |  | Davis et al. 1967 | Recorded elsewhere |
| 28 | *Gypsophila mollis* (Boiss.) Bornm*.* | Caryophyllaceae | *Bolanthus* hirsutus (Labill.) Barkoudah | *Saponaria hirsuta* Labill. | Syr, Lb, Eg (Sinai) |  | Barkoudah 1962 | Taxonomic revision |
| 29 | *Lamium ehrenbergii* Boiss. & Reut. | Lamiaceae | *Lamium purpureum* var. *ehrenbergii* (Boiss. & Reut.) Mennema |  | Tur, Syr, Lb |  | Davis et al. 1982 | Recorded elsewhere |
| 30 | *Linaria aucheri* Boiss. | Scrophulariaceae | *Linaria kurdica* subsp. *aucheri* (Boiss.) P.H.Davis |  | Tur, Syr, Lb |  | Davis et al. 1978 | Taxonomic revision |
| 31 | *Malus trilobata* (Labill. ex Poir.) C.K.Schneid. | Rosaceae |  | *Crataegus trilobata* Labill. ex Poir. | Gr, Bul, Tur, Lb, Pal | LC | Davis et al. 1972 | Recorded elsewhere |
| 32 | *Melissa inodora* Bornm. | Lamiaceae | *Melissa officinalis* subsp. *inodora* Bornm. |  | Tur, Lb |  | Davis et al. 1970 | Recorded elsewhere |
| 33 | *Onosma caerulescens* Boiss. | Boraginaceae |  |  | Tur, Syr, Lb, Pal, Irq, Irn |  | Davis et al. 1978 | Recorded elsewhere |
| 34 | *Orobanche hermonis* Mouterde | Orobanchaceae | *Orobanche camptolepis* Boiss. & Reut. |  | Tur, Syr, Lb, Jo, Irq, Irn, Sau, Cas |  | POWO 2025 | Taxonomic revision |
| 35 | *Orobanche libanotica* (Schweinfurth) G.Beck. | Orobanchaceae |  | *Phelypaea libanotica* Schweinf. ex Boiss. | Gr, Tur, Syr, Lb, Irq, Irn, Cau, Cas |  | Raab-Straube *et al.* 2018 | Taxonomic revision |
| 36 | *Poterium compactum* Boiss. | Rosaceae | *Sanguisorba minor* subsp. *lasiocarpa* (Boiss. & Hausskn.) Nordborg | *Poterium lasiocarpum* Boiss. & Hausskn. | Tur, Syr, Lb, Jo, Irq, Irn, Cau, Was |  | Davis et al. 1972 | Taxonomic revision |
| 37 | *Ranunculus myosuroides* Boiss. | Ranunculaceae |  |  | Tur, Syr, Lb, Pal |  | Davis et al. 1965 | Recorded elsewhere |
| 38 | *Rosularia kesrouanensis* Mouterde | Crassulaceae | *Rosularia sempervivum* subsp. *libanotica* (Labill.) Eggli |  | Tur, Syr, Lb | EN | Eggli 1988 | Taxonomic revision |
| 39 | *Rosularia parvifolia* Fröd. & Sam. ex Rech.f. | Crassulaceae | *Rosularia sempervivum* subsp. *libanotica* (Labill.) Eggli |  | Tur, Syr, Lb |  | Eggli 1988 | Taxonomic revision |
| 40 | *Scariola triquetra* (Labill.) Sojak | Asteraceae | *Astartoseris triquetra* (Labill.) N.Kilian, Hand, Hadjik., Christodoulou & Bou Dagh. | *Prenanthes triquetra* Labill. | Lb, Cyp | NT | Kilian et al. 2017 | Recorded elsewhere |
| 41 | *Silene damascena* Boiss. & Gaill. | Caryophyllaceae |  |  | Lb, Pal | LC | Danin & Fragman-Sapir 2016+ | Recorded elsewhere |
| 42 | *Silene grisea* Boiss. | Caryophyllaceae |  |  | Lb, Pal | VU | Danin & Fragman-Sapir 2016+ | Recorded elsewhere |
| 43 | *Sison exaltatum* Boiss. | Apiaceae |  |  | Lb, Syr, Pal |  | Danin & Fragman-Sapir 2016+ | Recorded elsewhere |
| 44 | *Thlaspi brevicaule* Boiss. & Ky | Brassicaceae | *Noccaea platycarpa* (Fisch. & C.A.Mey.) Al-Shehbaz | *Thlaspi platycarpum* Fisch., C.A.Mey. & N.Busch | Gr, Tur, Syr, Lb, Irq, Irn, Cau, Cas |  | Mao and Dash 2020 | Recorded elsewhere |
| 45 | *Trifolium billardieri* Sprengel | Fabaceae |  |  | Lb, Pal |  | Danin & Fragman-Sapir 2025 | Recorded elsewhere |
| 46 | *Tulipa aucheriana* Baker subsp. *westii* Mouterde | Liliaceae | *Tulipa humilis* var. *aucheriana* (Baker) Christenh. | *Tulipa aucheriana* Baker | Syr, Lb, Irn, Cau | LC | Christenhusz et al. 2013 | Taxonomic revision |
| 47 | *Tulipa lownei* Baker | Liliaceae | *Tulipa humilis* Herb. var. *humilis* |  | Tur, Syr, Lb, Pal, Cau, Irn | LC | Christenhusz et al. 2013 | Taxonomic revision |
| 48 | *Vicia canescens* Labill. | Fabaceae |  |  | Gr, Tur, Lb, Irq, Irn |  | Jalilian et al. 2014 | Recorded elsewhere |

References

Barkoudah YI (1962) A Revision of Gypsophila, Bolanthus, Ankyropetalum and Phryna. Wentia 9: 1–203. https://doi.org/10.1111/j.1438-8677.1962.tb00012.x

Bernardi L (1979) Tentamen revision generis Ferulago. Boissiera 30: 1–182. https://doi.org/10.5169/seals-895589

CHG (2025) Geneva Herbaria Catalogue. Available from: https://www.ville-ge.ch/musinfo/bd/cjb/chg/help.php?lang=en (January 20, 2025).

Christenhusz MJM, Govaerts R, David JC, Hall T, Borland K, Roberts PS, Tuomisto A, Buerki S, Chase MW, Fay MF (2013) Tiptoe through the tulips – cultural history, molecular phylogenetics and classification of Tulipa (Liliaceae). Botanical Journal of the Linnean Society 172: 280–328. https://doi.org/10.1111/boj.12061

Danin A, Fragman-Sapir O (2016+) Flora of Israel and adjacent areas. Flora of Israel and adjacent areas. Available from: https://flora.org.il:443/en/en/ (May 14, 2023).

Davis PH, Chamberlain DF, Matthews VA (1970) 3 Flora of Turkey and the East Aegean Islands, Volume 3. Edinburgh University Press, Edinburgh, UK.

Davis PH, Chamberlain DF, Matthews VA (1972) 4 Flora of Turkey and the East Aegean Islands, Volume 4. Edinburgh University Press, Edinburgh, UK.

Davis PH, Cullen J, Coode MJE (1965) 1 Flora of Turkey and the East Aegean Islands, Volume 1. Edinburgh University Press, Edinburgh, UK, 568 pp.

Davis PH, Cullen J, Coode MJE (1967) 2 Flora of Turkey and the East Aegean Islands, Volume 2. Edinburgh University Press, Edinburgh, UK, 580 pp.

Davis PH, Edmondson JR, Mill RR, Parris BS (1978) 6 Flora of Turkey and the East Aegean Islands, Volume 6. Edinburgh University Press, Edinburgh, UK, 826 pp.

Davis PH, Edmondson JR, Mill RR, Tan K (1982) 7 Flora of Turkey and the East Aegean Islands, Volume 7. Edinburgh University Press, Edinburgh, UK, 948 pp.

Davis PH, Edmondson JR, Mill RR, Tan K (1982) 7 Flora of Turkey and the East Aegean Islands, Volume 7. Edinburgh University Press, Edinburgh, UK, 948 pp.

Davis PH, Mill RR, Tan K (1984) 8 Flora of Turkey and the East Aegean Islands, Volume 8. Edinburgh University Press, Edinburgh, UK, 632 pp.

Eggli U (1988) A monographic study of the genus Rosularia (Crassulaceae). British Cactus & Succulent Society, Bury, UK, 119 pp.

Jalilian N, Rahiminejad MR, Maassoumi AA, Maroofi H (2014) Taxonomic revision of the genus Vicia L. (Fabaceae) in Iran. Iranian Journal of Botany 20: 155–164. https://doi.org/10.22092/ijb.2014.11010

Kilian N, Hand R, Hadjikyriakou GN, Christodoulou CS, Dagher-Kharrat MB (2017) Astartoseris (Cichorieae, Asteraceae), a new, systematically isolated monospecific genus accommodating Lactuca triquetra endemic to Lebanon and Cyprus. Willdenowia 47: 115–125. https://doi.org/10.3372/wi.47.47203

Mao AA, Dash SS (2020) 1 Flowering Plants of India: An Annotated Checklist, Volume 1: Dicotyledons. Botanical Survey of India, Kolkata, India, 970 pp.

Piwczyński M, Puchałka R, Spalik K (2015) The infrageneric taxonomy of Chaerophyllum (Apiaceae) revisited: New evidence from nuclear ribosomal DNA ITS sequences and fruit anatomy. Botanical Journal of the Linnean Society 178: 298–313.

Podlech D, Zarre S (2013) A Taxonomic Revision of the Genus Astragalus L. (Leguminosae) in the Old World. Naturhistorisches Museum Wien, Vienna, Austria, 2439 pp.

POWO (2025) Plants of the World Online. Plants of the World Online. Available from: http://www.plantsoftheworldonline.org/ (January 25, 2025).

Safikhani K, Mahmoodi M (2020) New record of Dianthus pendulus (Caryophyllaceae) from Iran. Iranian Journal of Botany 26: 19–21. https://doi.org/10.22092/ijb.2020.128607.1267
